# Inhibition of the canonical Wnt signaling pathway by a β-catenin/CBP inhibitor prevents heart failure by ameliorating cardiac hypertrophy and fibrosis

**DOI:** 10.1038/s41598-021-94169-6

**Published:** 2021-07-21

**Authors:** Thanachai Methatham, Shota Tomida, Natsuka Kimura, Yasushi Imai, Kenichi Aizawa

**Affiliations:** Division of Clinical Pharmacology, Department of Pharmacology, Jichi Medical University, 3311-1 Yakushiji, Shimotsuke-shi, Tochigi, 329-0498 Japan

**Keywords:** Cardiology, Molecular medicine

## Abstract

In heart failure (HF) caused by hypertension, the myocyte size increases, and the cardiac wall thickens. A low-molecular-weight compound called ICG001 impedes β-catenin-mediated gene transcription, thereby protecting both the heart and kidney. However, the HF-preventive mechanisms of ICG001 remain unclear. Hence, we investigated how ICG001 can prevent cardiac hypertrophy and fibrosis induced by transverse aortic constriction (TAC). Four weeks after TAC, ICG001 attenuated cardiac hypertrophy and fibrosis in the left ventricular wall. The TAC mice treated with ICG001 showed a decrease in the following: mRNA expression of brain natriuretic peptide (*Bnp)*, *Klf5*, fibronectin, β-MHC, and β-catenin, number of cells expressing the macrophage marker CD68 shown in immunohistochemistry, and macrophage accumulation shown in flow cytometry. Moreover, ICG001 may mediate the substrates in the glycolysis pathway and the distinct alteration of oxidative stress during cardiac hypertrophy and HF. In conclusion, ICG001 is a potential drug that may prevent cardiac hypertrophy and fibrosis by regulating KLF5, immune activation, and the Wnt/β-catenin signaling pathway and inhibiting the inflammatory response involving macrophages.

## Introduction

Heart failure (HF) is a significant health problem that continues to have high mortality. The disability caused by HF presents a substantial burden in society and affects the healthcare system^1^. Hypertension, myocardial infarction (MI), ischemia, and vascular disease cause an increase in myocyte size and cardiac wall thickness, indicating hypertrophy^2^. The heart compensates by increasing its size, but when cardiac hypertrophy exists, it sometimes leads to maladaptation.

In pathologic cardiac fibrosis, cardiac fibroblasts are the dominant collagen-generating cell type. Cardiac fibrosis caused by cardiac injury could disrupt cardiac conduction, reduce cardiac output, and increase fibrillar collagen, resulting in HF^3–5^. The accumulation of extracellular matrix (ECM) produced by cardiac fibroblasts is an influential modulator of the heart’s growth and cell function; however, excess ECM proteins deposited in the myocardium form a network of proteins around the cells, causing fibrosis that eventually promotes cardiac tissue stiffening and cardiac function decline, thereby damaging the cardiac structure and function^6–8^. Krüppel-like factor 5 (KLF5), a zinc-finger transcription factor that can be a molecular marker of myofibroblasts, has been found to phenotypically modulate smooth muscle cells^9^. KLF5 is important in the development and maintenance of the heart, aorta, and lung system^10^. Transverse aortic constriction (TAC) induces the upregulation of KLF5 expression and KLF5-controlled cardiac fibroblasts, which are involved in the myocardial adaptive response to pressure overload^11,12^. Moreover, KLF5 promotes cardiac hypertrophy and regulates peroxisome proliferator-activated receptor alpha (PPAR-α) expression^13,14^. Considering its relationship with cardiac inflammation and hypertrophy, PPAR-α has been gaining substantial attention^15^. A previous study showed that wild-type chronic pressure-overloaded mice treated with fenofibrate exhibited improved cardiac remodeling^16^. Fenofibrate has been used in treating dyslipidemia and hypertriglyceridemia, with PPAR-α activation reducing lipids^16^. In animal studies, PPAR-α inactivation may consequently change the phenotype and cardiac growth caused by pressure overload. Thus, compromised PPAR-α activity may be related to the progression from compensated left ventricular (LV) hypertrophy to HF in hypertensive heart disease^17,18^.

When the inflammatory cytokine response is activated and prolonged, deleterious cardiac effects persist, leading to the progression of LV dysfunction and HF^19^. The activation and accumulation of macrophages in the heart indicate the development of the innate immune response^20^. In the heart of mice with TAC, the inflammatory genes and inflammatory cells infiltrate^21,22^. The release of proinflammatory cytokines and growth factors by macrophages plays an important role in LV inflammation and remodeling. In patients with hypertension, the number of circulating proinflammatory monocytes and cytokines increases, and it continues to rise during symptomatic HF. This expansion in cardiac macrophages indicates HF progression. Myocardial monocyte infiltration and macrophage accumulation generally remain unknown until HF symptoms have developed in patients and mice with hypertension^23^. Macrophages and T cells in the heart accumulate within days to weeks after TAC occurrence; this phenomenon is linked to fibrosis and adverse ventricular remodeling^24–28^.

The Wnt/β-catenin pathway is one of the key mechanisms in HF pathogenesis, and an evolutionarily conserved signaling pathway of Wnt/β-catenin is involved in injury repair, organ development, inflammation, and tissue remodeling^29–32^. The Wnt/β-catenin signaling system plays an important role in the cardiac development and orchestration of a cardiac injury response^33^. A small-molecule inhibitor known as ICG001 impedes β-catenin–mediated gene transcription. When ICG001 is administered in rats, the β-catenin–mediated transcription is inhibited by improving the ejection fraction (EF) at 10 days post-MI^34^. In a recent study, β-catenin inhibition by ICG001 could protect both the heart and kidney in patients with cardiorenal syndrome^35^. However, the mechanisms of ICG001 to prevent HF are still poorly understood.

In this study, we demonstrated that ICG001 prevents and improves the heart against hypertrophy and dysfunction by reducing the fibrosis and macrophage accumulation and allows substrate metabolism alteration after pressure overload. Thus, ICG001 may have protective effects against cardiac hypertrophy and fibrosis via the regulation of KLF5, immune activation, and the Wnt/β-catenin signaling pathway and may also inhibit the inflammatory response involving macrophages.

## Results

### ICG001 increased the survival of mice with HF and improved their cardiac function after TAC

**Figure 1:**
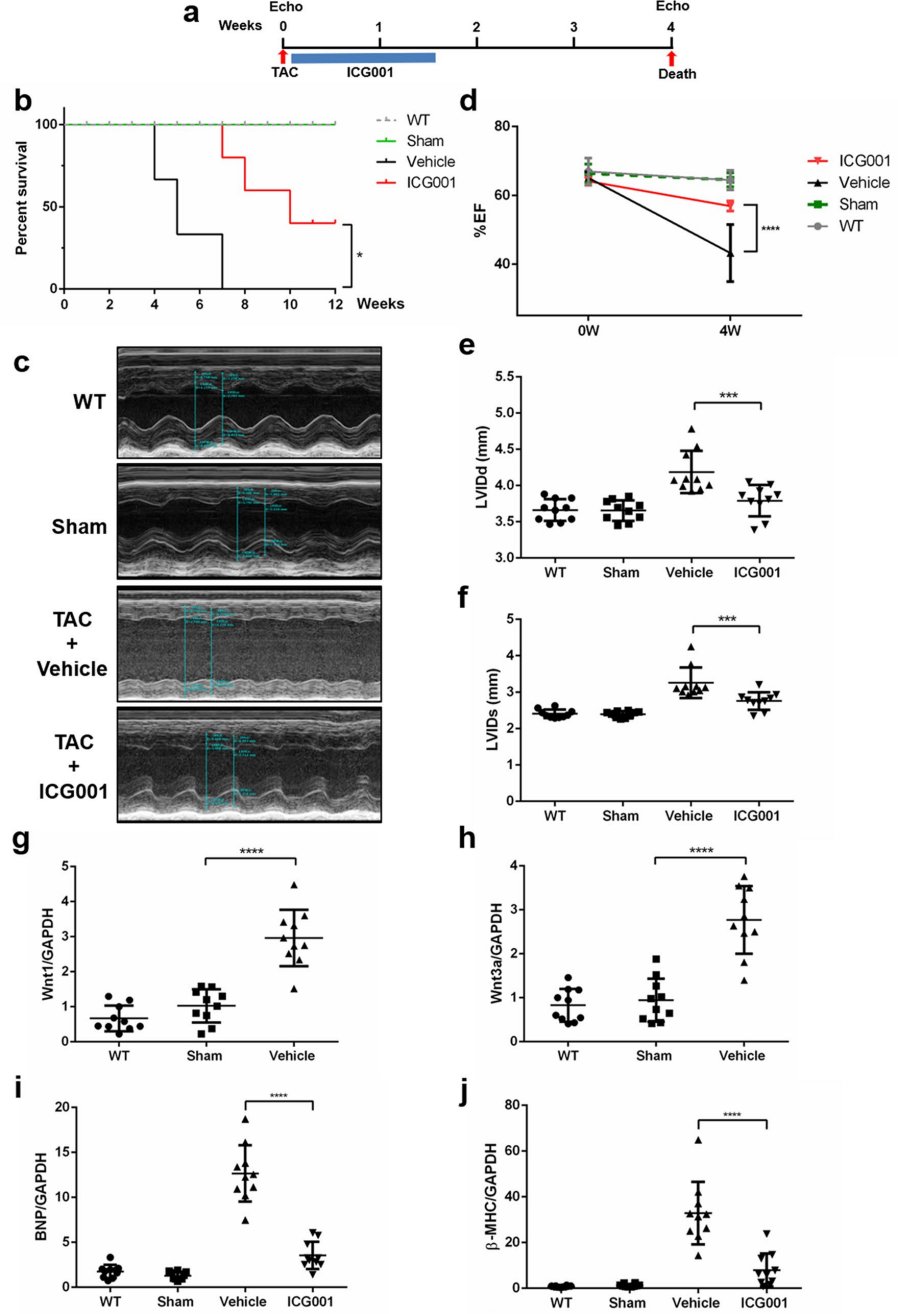
ICG001 increased the survival of mice with HF and improved the cardiac function after TAC. (**a**) Experimental design. (**b**) Long-term survival curves showed that the mortality rate was significantly ameliorated in ICG001-treated TAC mice compared with that in TAC mice after TAC; *P* < 0.05 (n = 10 per group). (**c**) Example of M-Mode echocardiography of the left ventricle (LV) at 4 weeks after TAC. (**d**) Left ventricular ejection fraction (LVEF) before TAC and at 4 weeks after TAC (%), (**e**) left ventricular internal dimension at end-diastole (LVIDd), and (**f**) left ventricular internal dimension at end-systole (LVIDs) at 4 weeks after TAC showed systolic dysfunction, and ICG001 improved cardiac function after TAC (n = 10). (**g**,**h**) Quantitative real-time polymerase chain reaction (PCR) showed that the genes *Wnt1* and *Wnt3a* were induced in the heart of mice for 4 weeks after TAC (n = 10). (**i**,**j**) Quantitative real-time PCR revealed that the expression of brain natriuretic peptide (*Bnp*) and β-myosin heavy chain (β-MHC) was induced after TAC and but then reduced by ICG001 treatment (n = 10). Statistical significance of distributed data was analyzed by one-way and two-way ANOVA followed by Tukey’s multiple comparisons test. ****P < 0.0001, ***P < 0.001, *P < 0.05 vs. vehicle group.

We used the TAC mouse model to induce LV pressure overload that causes cardiac hypertrophy and HF. From 10 days to 1 month after TAC occurrence, few TAC mice died. Five hours after TAC, the mice were injected with ICG001 (50 mg/kg/day) intraperitoneally for 10 days (twice per day) and classified as the ICG001-treated TAC mouse group (Fig. 1a). Up to 4 weeks after TAC occurrence, the untreated TAC mice started to die because of acute HF caused by pressure overload, and within 7 weeks after TAC, all of them died. Meanwhile, those that received ICG001 treatment survived up to 12 weeks (Fig. 1b). Hence, ICG001 treatment significantly improved the survival of mice with HF induced by the established LV pressure overload. Moreover, the cardiac function was evaluated by echocardiography (Fig. 1c). Four weeks after TAC, the EF was significantly higher in the ICG001-treated TAC mouse group than in the vehicle group (Fig. 1d). Echocardiography also demonstrated that the left ventricular internal dimension at end-diastole (LVIDd) and left ventricular internal dimension at end-systole (LVIDs) were significantly higher in TAC mice than in ICG001-treated TAC mice (Fig. 1e,f, Table 1). These results indicate that ICG001 prevented the decrease of the cardiac function after TAC. Furthermore, the Wnt ligands *Wnt1* and *Wnt3a* increased in 4 weeks in TAC mice (Fig. 1g,h). Thus, Wnt/β-catenin was activated in the heart after TAC. The mRNA expression levels of the HF markers, namely, brain natriuretic peptide (*Bnp*) and β-myosin heavy chain (β-MHC), were significantly lower in ICG001-treated TAC mice than in TAC mice (Fig. 1i,j). Therefore, the ICG001 treatment improved the cardiac function after TAC.

**Table 1:** Echocardiographic measurements in mice of each group.

Groups	WT (n = 10)	Sham (n = 10)	TAC (n = 10)	TAC + ICG001 (n = 10)
IVSd (mm)	0.75 ± 0.08	0.76 ± 0.03	1.38 ± 0.16^#^	0.79 ± 0.09^#^
IVSs (mm)	1.09 ± 0.13	1.05 ± 0.09*	0.93 ± 0.06^#^	1.20 ± 0.10^#^
LVIDd (mm)	3.66 ± 0.15	3.65 ± 0.14	4.19 ± 0.29^#^	3.79 ± 0.22^#^
LVIDs (mm)	2.41 ± 0.11	2.39 ± 0.09*	3.26 ± 0.42^#^	2.76 ± 0.24^#^
LVPWd (mm)	0.76 ± 0.08	0.74 ± 0.04	0.97 ± 0.17^#^	0.88 ± 0.13^#^
LVPWs (mm)	1.15 ± 0.10	1.09 ± 0.12	1.30 ± 0.09^#^	1.20 ± 0.16^#^
LVEDV (μl)	59.46 ± 7.20	58.08 ± 6.39	89.18 ± 10.85^#^	63.41 ± 6.69^#^
LVESV (μl)	21.18 ± 3.47	20.44 ± 2.01*	46.69 ± 6.48^#^	29.59 ± 5.52^#^
LVM (corrected) (mg)	82.66 ± 7.25	76.32 ± 9.21*	113.11 ± 10.60^#^	93.91 ± 13.30^#^
LVEF (%)	64.44 ± 2.83	64.73 ± 1.71*	45.78 ± 5.72^#^	57.17 ± 1.72^#^
LVFS (%)	34.55 ± 2.07	34.58 ± 1.46*	22.13 ± 3.30^#^	29.57 ± 1.06^#^
HR (bpm)	529.5 ± 11.79	528.7 ± 11.99	565.1 ± 11.26^#^	531.1 ± 8.94^#^

Statistical significance of distributed data was analyzed by one-way ANOVA followed by Tukey’s multiple comparisons test (n = 10 per group).

*IVSd* interventricular septum depth at end-diastole; *IVSs* interventricular septum depth at end-systole; *LVIDd* left ventricular internal diameter at end-diastole; *LVIDs* left ventricular internal dimension at end-systole; *LVPWd* left ventricular posterior wall depth at end-diastole; *LVPWs* left ventricular posterior wall depth at end-systole; *LVEDV* left ventricular end-diastolic volume; *LVESV* left ventricular end-systolic volume; *LVM* left ventricular mass (corrected); *LVEF* left ventricular ejection fraction; *LVFS* left ventricular fractional shortening; *HR* heart rate; *bpm* beats per minute.

*P < 0.05 sham vs. ICG001.

^#^*P* < 0.05 vehicle (untreated TAC mice) vs. ICG001.

### ICG001 attenuated cardiac hypertrophy in vivo and ameliorated cardiac fibrosis induced after TAC

**Figure 2:**
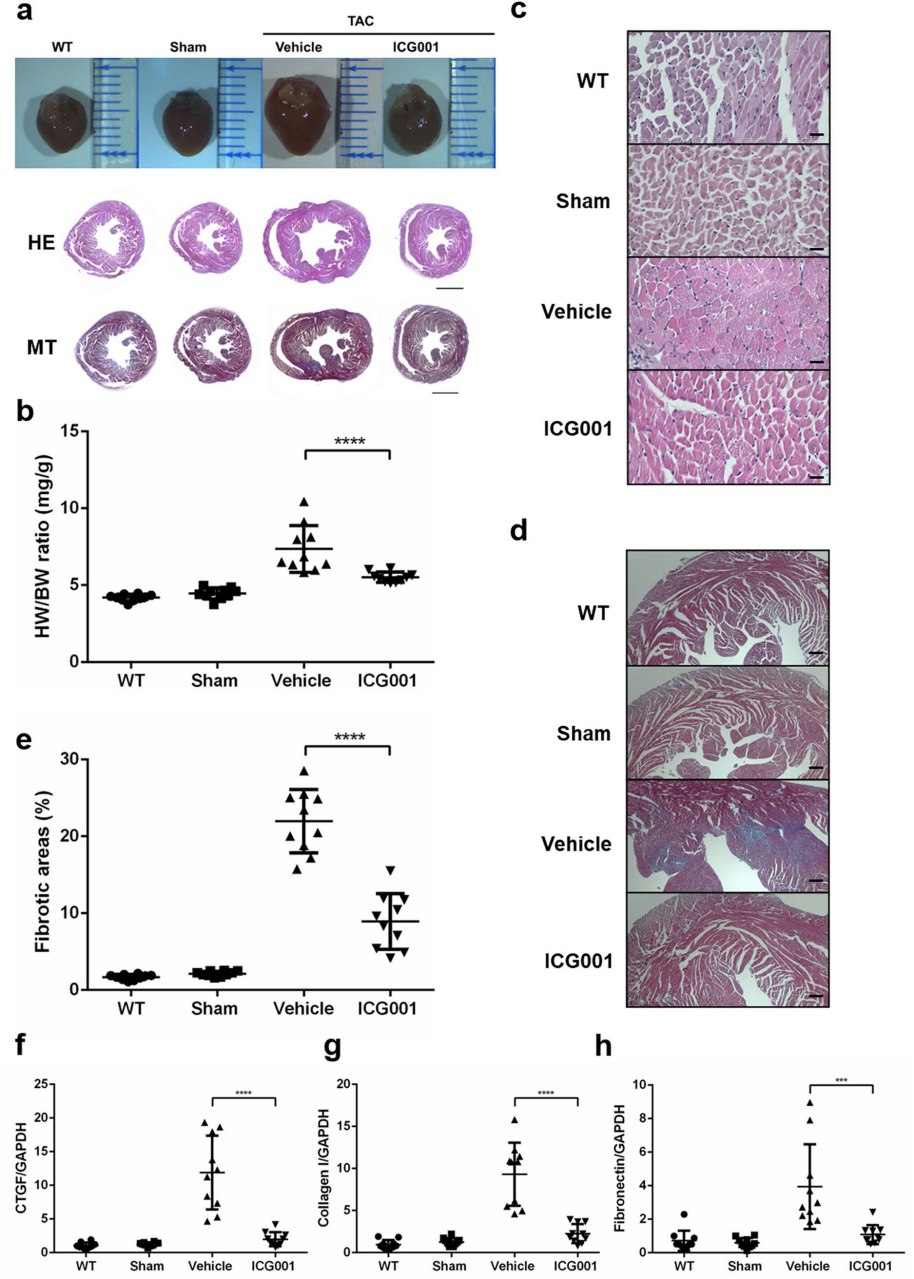
ICG001 attenuated cardiac hypertrophy in vivo and ameliorated cardiac fibrosis induced after TAC. (**a**) Representative images of heart size (upper panel), hematoxylin and eosin (HE) staining (middle panel), and Masson’s trichrome (MT) staining (bottom panel) of cross sections of hearts from WT, sham, TAC mice, and ICG001-treated TAC mice at 4 weeks after TAC. Scale bar: 2 mm. (**b**) Heart weight-to-body weight (HW/BW) ratio (n = 10). (**c**) Heart slices were histologically analyzed by HE staining. Scale bar: 200 µm. (**d**) MT staining. Scale bar: 200 µm. (**e**) Statistical results for the fibrotic areas in the indicated group (n = 10). (**f–h**) Relative connective tissue growth factor (*Ctgf*), collagen I, and fibronectin levels in the LVs of mice from the indicated groups (n = 10). Statistical significance of distributed data was analyzed by one-way ANOVA followed by Tukey’s multiple comparisons test. ****P < 0.0001, ***P < 0.001 vs. vehicle group.

Unlike the sham mice, the TAC mice exhibited cardiac hypertrophy, as demonstrated by the increase in heart weight/body weight (HW/BW) ratio at the end of 4 weeks. The morphological changes and hematoxylin and eosin (HE) staining of heart sections also revealed a hypertrophic change in TAC mice while a markedly diminished hypertrophic response to pressure overload in ICG001-treated TAC mice (Fig. 2a–c). Therefore, ICG001 attenuated cardiac hypertrophy after TAC. Furthermore, we measured the cardiomyocyte sizes. We found that ICG001-treated TAC mice had significantly smaller cardiomyocytes than vehicle-treated TAC mice (Supplementary Fig. 1). Moreover, Masson’s trichrome (MT) staining showed that the interstitial fibrosis in cardiac tissues increased in TAC mice, but this increase was alleviated in ICG001-treated TAC mice (Fig. 2d,e). The mRNA expression levels of several cardiac fibrosis markers, including connective tissue growth factor (*Ctgf*), collagen I, and fibronectin, were markedly lower in the ICG001-treated TAC mouse group than in the vehicle group (Fig. 2f–h). Thus, ICG001 ameliorated the cardiac fibrosis after TAC.

### ICG001 induced protein expression of cardiac hypertrophy and fibrosis after TAC

**Figure 3:**
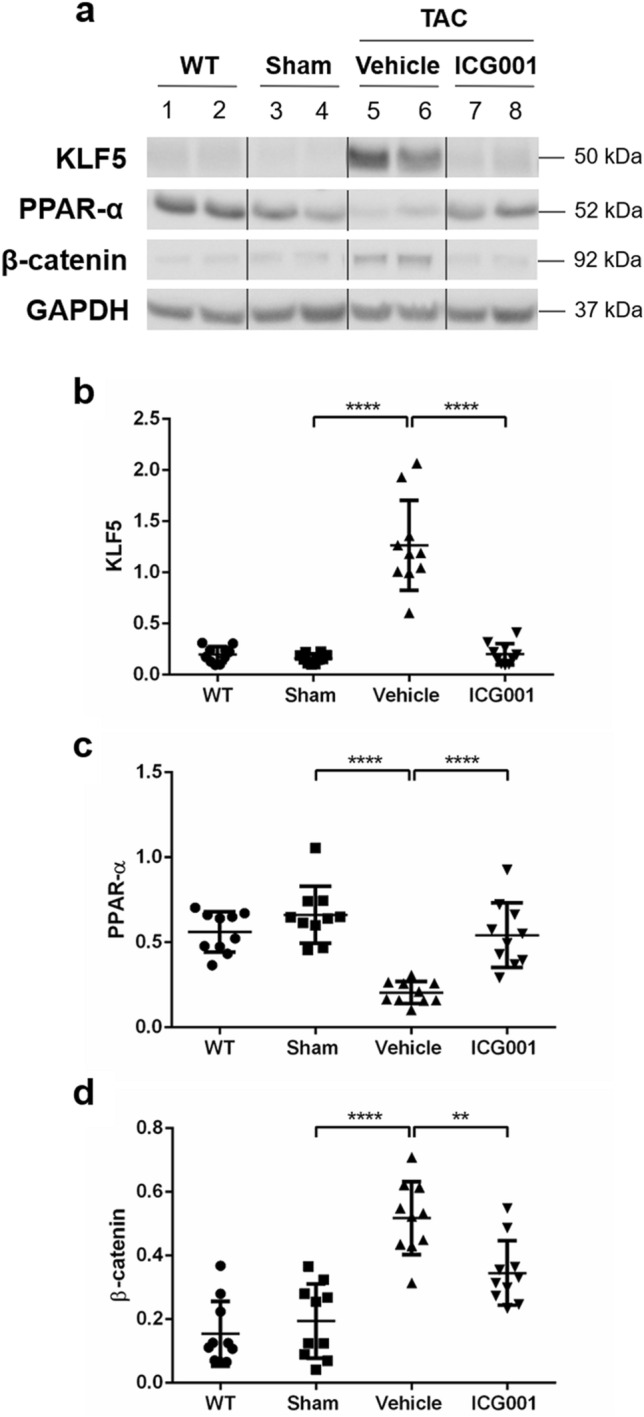
ICG001 induced gene and protein expression of cardiac hypertrophy and fibrosis after TAC. (**a**) Western blot analyses showed the protein expression of KLF5, PPAR-α, and β-catenin in the heart of mice from various groups, as indicated. GAPDH was used as a loading control. (**b–d**) Quantitative data of the protein levels of KLF5, PPAR-α, and β-catenin are presented in various groups, as indicated (n = 10). The vertical lines indicate the grouping of cropped images. Statistical significance of distributed data was analyzed by one-way ANOVA followed by Tukey’s multiple comparisons test. ****P < 0.0001, **P < 0.01 vs. vehicle group.

We next investigated whether ICG001 is associated with the roles of KLF5 and PPAR-α in responding cardiac hypertrophy and fibrosis. The expression of KLF5 protein was significantly upregulated in TAC mice but was significantly reduced after ICG001 treatment (Fig. 3a,b). Furthermore, the expression of PPAR-α protein was decreased in TAC mice but was significantly increased after ICG001 treatment (Fig. 3c). We also found that cardiac β-catenin protein was activated in TAC mice but was significantly suppressed in ICG001-treated TAC mice (Fig. 3d). Thus, ICG001 inhibits cardiac β-catenin activation in Wnt/β-catenin signaling and might have an impact on protective effects against cardiac hypertrophy and fibrosis via KLF5 and PPAR-α.

### ICG001 attenuated macrophage accumulation in cardiac tissues after TAC

**Figure 4:**
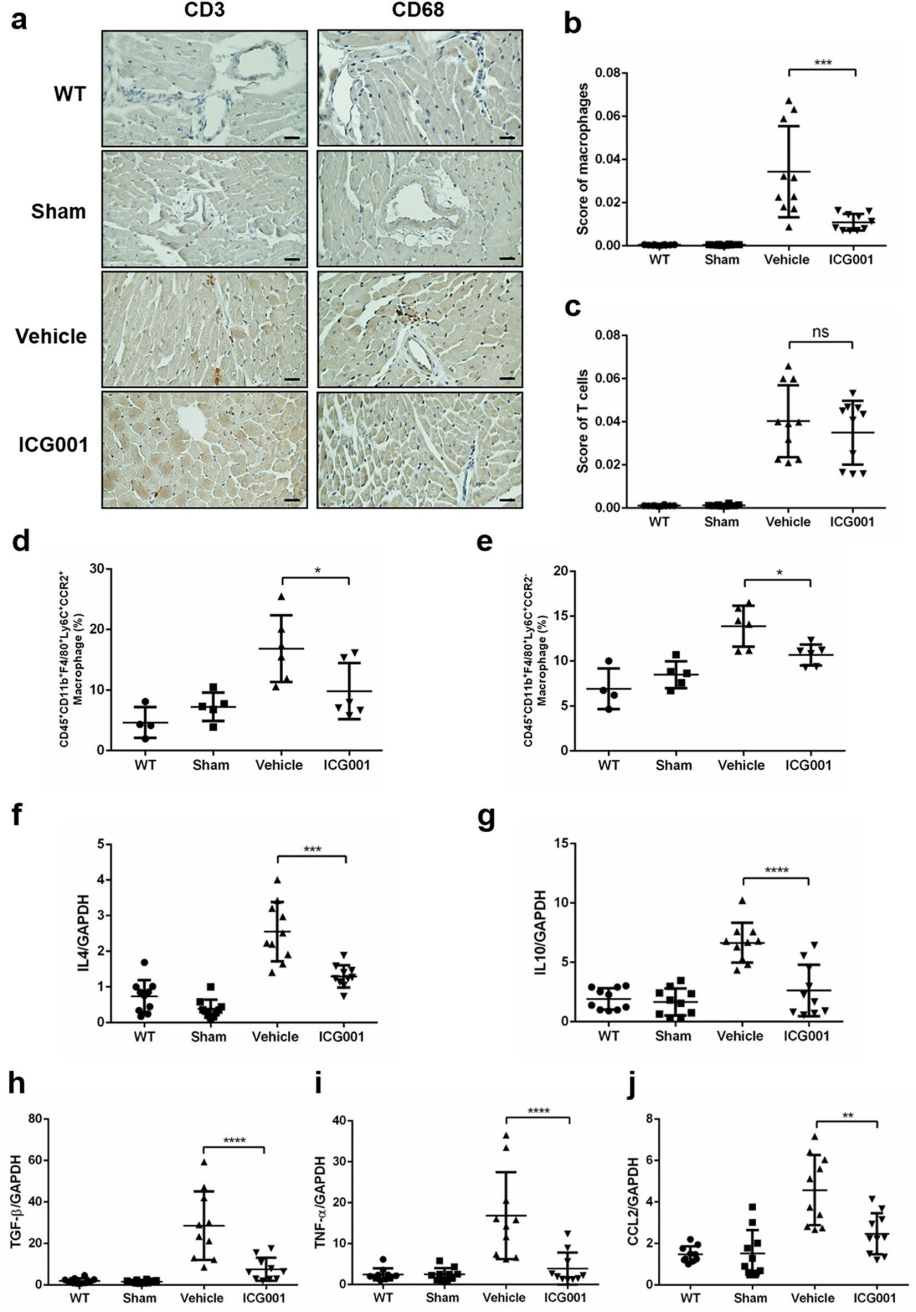
ICG001 attenuated macrophage accumulation in cardiac tissues after TAC. (**a**) Immunohistochemical analysis of CD3 and CD68 in heart sections (n = 10, Scale bar = 200 µm). (**b**,**c**) Score of macrophages detected by CD68 and T cells detected by CD3 in immunohistochemical analysis calculated by ImageJ^61^ (ImageJ version 1.53e) (n = 10). (**d**,**e**) Flow cytometry analysis of the macrophages CD45^+^CD11b^+^F4/80^+^Ly6C^+^CCR2^+^ and CD45^+^CD11b^+^F4/80^+^Ly6C^+^CCR2^−^ in mice at 4 weeks after TAC (n = 4–6). (**f**–**j**) Quantitative real-time polymerase chain reaction of *Il4*, *Il10*, *Tgfb1*, *Tnfa*, and *Ccl2* in the heart at 4 weeks after TAC (n = 10). Statistical significance of distributed data was analyzed by one-way ANOVA followed by Tukey’s multiple comparisons test. *ns* not significant, ****P < 0.0001, ***P < 0.001, **P < 0.01, *P < 0.05 vs. vehicle group.

Inflammation has been known to precede cardiac fibrosis in TAC mice^36^; therefore, we investigated the accumulation of T cells and macrophages in the heart after TAC. In the immunohistochemical analysis of CD3 and CD68 in heart sections, the infiltration of T cells and macrophages occurred in TAC mice, and they were both observed in the cardiac tissues after TAC (Fig. 4a). In TAC mice treated with ICG001, the macrophages detected by CD68 significantly decreased (Fig. 4b), but the T cells detected by CD3 did not (Fig. 4c). Furthermore, flow cytometry was performed to identify cardiac macrophages. In ICG001-treated TAC mice, the level of both CD45^+^CD11b^+^F4/80^+^Ly6C^+^CCR2^+^ and CD45^+^CD11b^+^F4/80^+^Ly6C^+^CCR2^−^ macrophages infiltrating in the aortic wall significantly decreased (Fig. 4d,e). Hence, ICG001 reduced both the tissue resident cardiac macrophages CCR2^+^ and CCR2^−^ after TAC. The ICG001 treatment also led to a marked reduction in transcripts that encode inflammatory mediators including interleukin 4 (*Il4*), interleukin 10 (*Il10*), transforming growth factor beta 1 (*Tgfb1*), tumor necrosis factor alpha (*Tnfa*), and chemokine (C–C motif) ligand 2 (*Ccl2*) in the heart at 4 weeks after TAC (Fig. 4f–j). Thus, ICG001 might regulate cardiac inflammation by influencing the macrophages and reducing their accumulation after TAC.

### ICG001 mediated the substrate metabolism and metabolic flexibility in TAC mice

**Figure 5:**
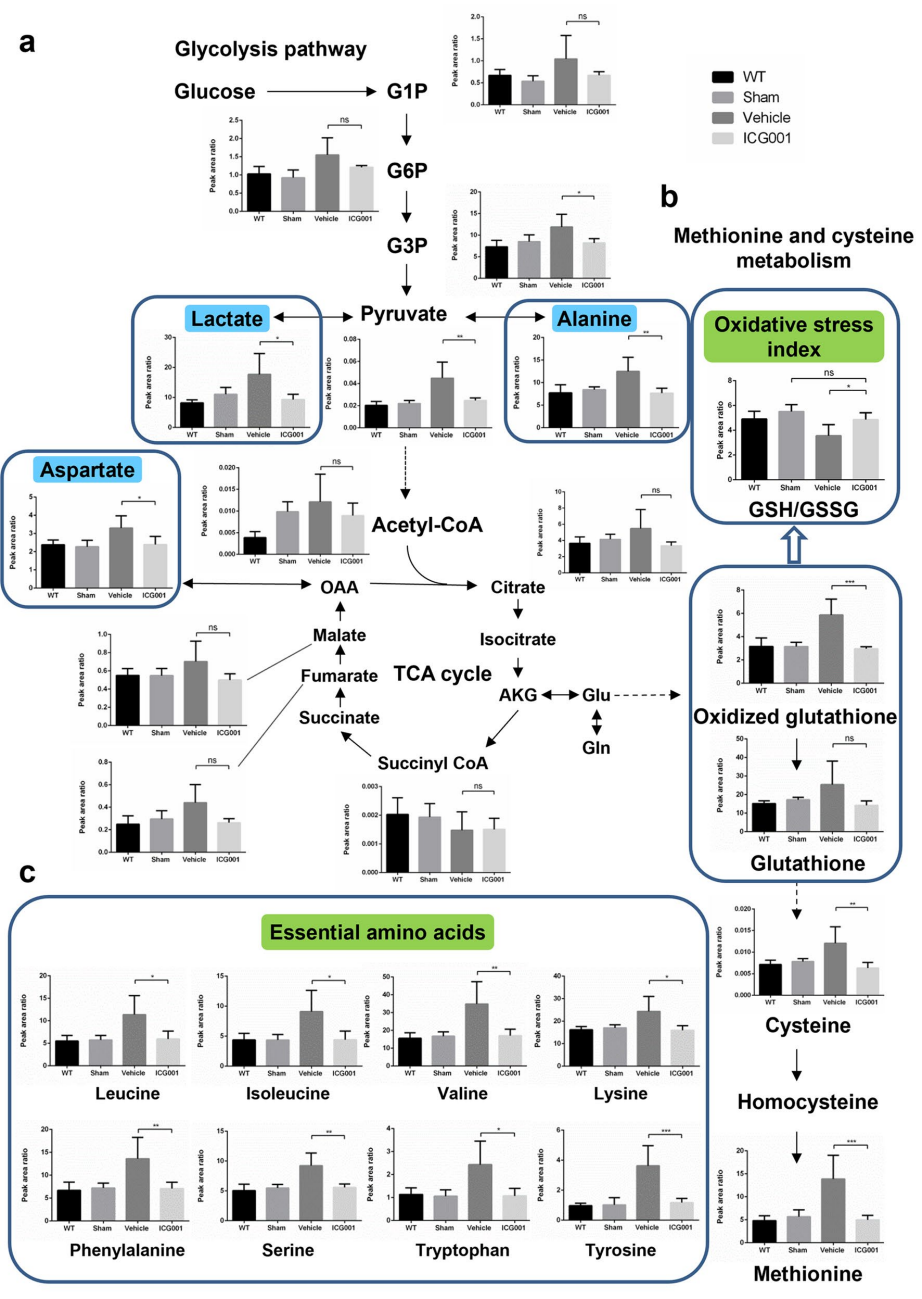
Substrate metabolism and metabolic flexibility in ICG001-treated TAC mice. Data were normalized to an internal control when the unit is indicated by area ratio. (**a**) Metabolic profiling in the glycolysis pathway and the TCA cycle of heart tissues from mice in the indicated group (n = 4–5). Lactate, aspartate, and alanine levels were significantly lower in the ICG001-treated TAC mice (blue squares) compared to the vehicle-treated TAC mice. (**b**) Metabolic profiling in methionine and cysteine showed the oxidative stress index calculated from the ratio of reduced (GSH) and oxidized glutathione (GSSG) (GSH/GSSG) (n = 4–5). (**c**) Essential amino acid levels in heart tissues from mice in the indicated group (n = 4–5). Statistical significance of distributed data was analyzed by one-way ANOVA followed by Tukey’s multiple comparisons test. *ns* not significant, ***P < 0.001, **P < 0.01, *P < 0.05 vs. vehicle group. *G1P* glucose-1-phosphate, *G6P* glucose-6-phosphate, *G3P* glycerol-3-phosphate, *AKG* alpha-ketoglutarate, *Glu* glutamic acid, *Gln* glutamine, *OAA* oxaloacetic acid.

We investigated the impact of hypertrophy and HF induced by pressure overload on metabolism. In the glycolysis pathway, the levels of glucose-1-phosphate (G1P) and glucose-6-phosphate (G6P) were higher in TAC mice than in sham; however, in ICG001-treated TAC mice, both levels decreased but were not statistically significant. Meanwhile, glycerol-3-phosphate (G3P) was increased in TAC mice but was reduced in ICG001-treated TAC mice. Compared with sham and ICG001-treated TAC mice, TAC mice had increased levels of pyruvate, alanine, and lactate in the glycolysis pathway and demonstrated minor changes in tricarboxylic acid cycle (TCA) intermediates such as citrate, fumarate, and malate after TAC. Moreover, aspartate was increased after TAC, but it was significantly decreased in ICG001-treated TAC mice. Regarding methionine and cysteine metabolisms, methionine increased after TAC but decreased after ICG001 treatment. Additionally, the indices of oxidative stress were assessed according to the ratio of reduced (GSH) and oxidized glutathione (GSSG). Compared with that in sham, the GSH/GSSG ratio was slightly decreased in ICG001-treated TAC mice but further decreased in TAC mice (Fig. 5a,b). Taken together, ICG001 may mediate the substrates in the glycolysis pathway and the distinct alteration of oxidative stress during cardiac hypertrophy and HF. Moreover, the metabolites and amino acids including leucine, isoleucine, valine, lysine, phenylalanine, serine, tryptophan, and tyrosine increased in TAC mice but were significantly attenuated in ICG001-treated TAC mice (Fig. 5c).

## Discussion

This study showed that ICG001 prevented HF and identified the molecular mechanisms and cellular response that led to cardiac function improvement and fibrosis attenuation. We also obtained a clinically important result, that is, starting ICG001 treatment before the onset of HF improves the survival rate of TAC mice.

Injection of ICG001 to the TAC mice attenuated several pathogenic changes in the heart, thereby preventing HF. Echocardiography indicated that the pressure overload did not impair the cardiac function of ICG001-treated mice. The low expression of *Bnp,* which is a well-established marker of HF, also suggests that ICG001 treatment prevented HF. The ICG001-treated TAC mice had lower β-MHC expression, thinner LV wall (Table 1), and lower HW/BW ratio, indicating that they did not develop cardiac hypertrophy, which generally precedes HF. Fibrosis is found in both the hypertrophic heart and failing heart^37^. Fibroblast-specific staining showed that ICG001 also attenuated interstitial fibrosis. The upregulation of Wnt1 and Wnt3a in TAC mice indicated the activation of Wnt/β-catenin signaling^35^. Indeed, pressure overload increased the expression of β-catenin as well as Wnt1 and Wnt3a, and the ICG001 treatment in TAC mice lowered the β-catenin expression back to the baseline. Given that the Wnt/β-catenin signaling is indispensable for the expression of excessive ECM gene and deposition of collagen after pressure overload^38^, the decrease in both the expression and activity of β-catenin may contribute to the lowering of the expression of *Ctgf*, collagen I, and fibronectin, thereby preventing fibrosis. Furthermore, the survival rate of the ICG001-treated TAC mice was significantly higher than the vehicle-treated TAC mice. These results indicated that ICG001 prevented HF by improving cardiac function and inhibiting cardiac hypertrophy and cardiac fibrosis, leading to an improved prognosis.

Pressure overload upregulates KLF5, which promotes cardiac hypertrophy and proliferates cardiac fibroblasts^11,12^. In the present study, ICG001 inhibited KLF5 upregulation induced by pressure overload. Considering that *Klf5* haploinsufficiency decreases M1 macrophage accumulation^39^, we tested whether TAC-induced pressure overload in mice activated the accumulation of inflammatory cells in cardiac tissues. Both of the T cell and macrophage infiltrations were observed in cardiac tissues after TAC. Importantly, macrophage infiltration was suppressed after ICG001 treatment; however, T cell infiltration was not affected in ICG001-treated mice, which indicated that ICG001 only attenuated macrophage infiltration. Furthermore, transcripts that encode inflammatory mediators such as *Il4*, *Il10*, *Tgfb1*, *Tnfa*, and *Ccl2* in the heart after TAC were markedly reduced in ICG001-treated TAC mice. Since CCL2 is chemotactic to monocytes^40^, CCL2 reduction by ICG001 may have minimized the recruitment of monocytes, resulting in macrophage decrease in the heart. Reducing the expression of inflammatory cytokines such as IL-4, IL-10, and TNFα inhibits the pressure overload-induced cardiac dysfunction or attenuation of cardiac hypertrophy and fibrosis in mice^41,42^; hence, the reduction of such cytokines correlated with ICG001 injection may also contribute to HF prevention. In addition, fibroblasts activated by macrophages produce ECM proteins^43^. Thus, reduction in macrophage accumulation after ICG001 treatment may also contribute to fibrosis attenuation.

Time-course studies previously revealed that the number of cardiac macrophages moderately increases after TAC, reaching its peak at 7 days, and then decreases to baseline after 2 weeks^44^. Considering that the cardiac resident macrophage proliferation occurred within the first week after pressure overload, reducing macrophage proliferation in the early phase may be important for cardiac repair and HF prevention. In our experiment, ICG001 was injected to the mice for 10 days in the early period after TAC. ICG001 could reduce the macrophage accumulation after TAC. Macrophage reduction by ICG001 administration prevented HF in TAC mice. However, the timing of ICG001 injection to prevent macrophage accumulation may be crucial in achieving HF prevention in TAC mice. Our finding suggested that ICG001 may prevent further macrophage proliferation in the early stage or later remodeling phase and has a potential effect to prevent the macrophage accumulation in TAC mice.

**Figure 6:**
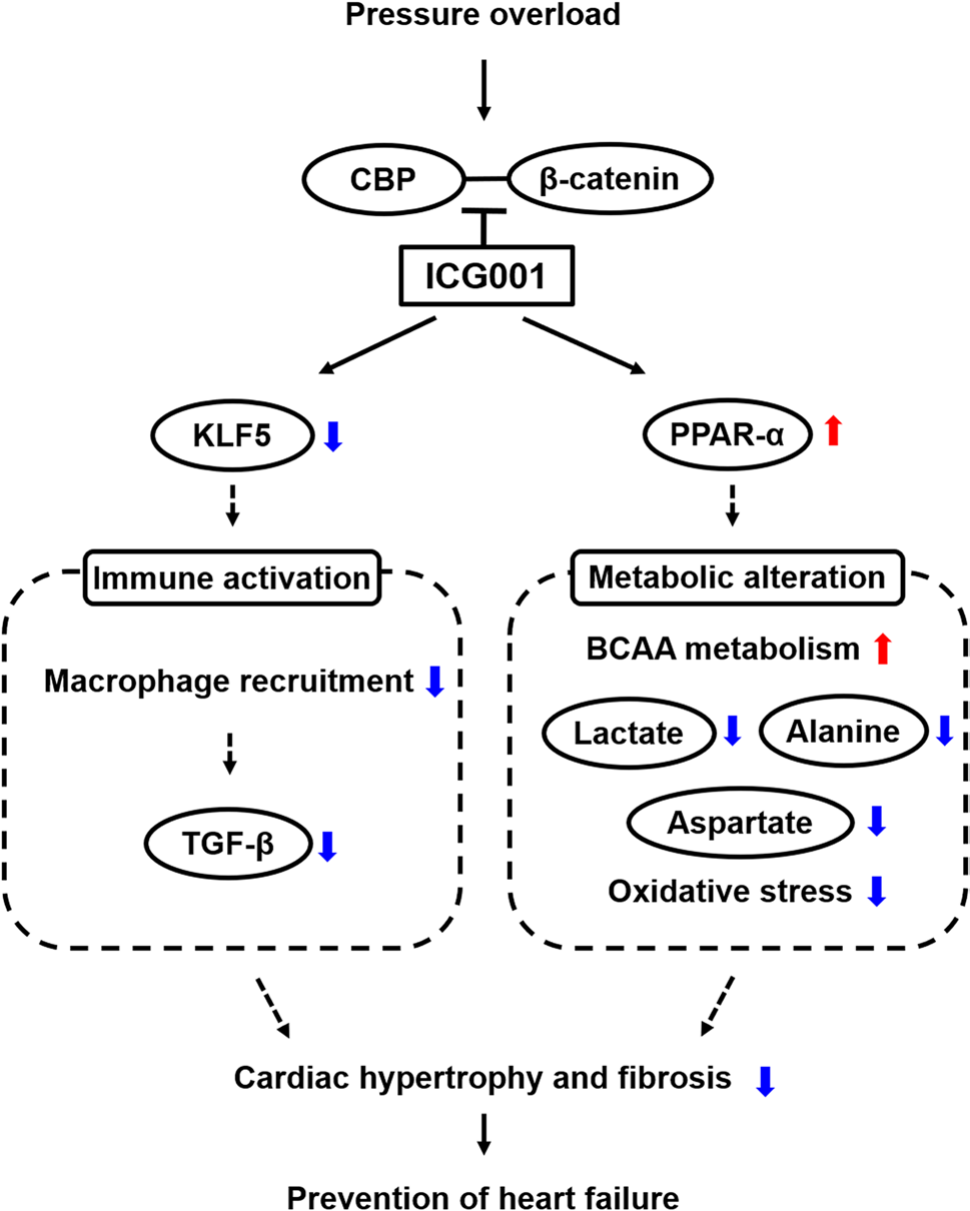
Summary of the proposed pathways of the role of ICG001 to attenuate cardiac hypertrophy and ameliorate cardiac fibrosis induced by pressure overload. ICG001 prevented heart failure induced by pressure overload possibly through two pathways. The dotted lines indicate the hypothesized chain of event. One of the pathways attenuated immune activation mediated by KLF5 upregulation, while the other pathway blocked the PPAR-α downregulation that causes metabolic alteration. ICG001 inhibited the binding of CREB-binding protein (CBP) to β-catenin and then reduced the expression of KLF5 leading to the reduction of macrophage recruitment and TGF-β. Inhibition of CBP and β-catenin complex formation by ICG001 prevented accumulation of lactate, aspartate, and alanine and improved the metabolism of branched-chain amino acid (BCAA). Oxidative stress was reduced by ICG001 according to the ratio of reduced and oxidized glutathione. Both pathways ameliorated cardiac hypertrophy and fibrosis and ultimately prevented HF.

Considering that ICG001 could reduce TGF-β, TGF-β might be the mediator in the regulation of KLF5, immune activation, and the Wnt/β-catenin signaling pathway. The upregulation of TGF-β is persistent for at least 4–8 weeks when fibronectin and collagen are expressed in cardiac tissues^45^. TGF-β is directly controlled by KLF5 and involved in the development of fibrosis in various chronic inflammatory conditions^46^. However, in the present study, TGF-β signaling also participated in the activation of the β-catenin-dependent pathway, and the Wnt/β-catenin signaling pathway played a role in inducing TGF-β signaling. The key regulators of myofibroblast biology in cardiac fibrosis were observed in the TGF-β and Wnt signaling pathways. The previous study had shown that blocking these pathways prevented fibrosis^47^. In addition, the overexpression of FGF23 in cardiac fibroblasts promotes fibroblast proliferation through β-catenin signaling activation and TGF-β upregulation^48^. Moreover, macrophages secrete TGF-β1, IL-10, and ECM proteins in cardiac fibrosis to activate fibroblasts^43^. The aforementioned studies suggest that TGF-β plays a central role in the development of fibrosis between KLF5, immune activation, and the Wnt/β-catenin signaling pathway. We propose that ICG001 may control pathological cardiac hypertrophy and fibrosis via KLF5, immune activation, and the Wnt/β-catenin signaling pathway by connecting with TGF-β as a mediator (Fig. 6). Moreover, ICG001 may regulate macrophage accumulation that influences fibrosis infiltration continually.

The current study also found that the expression of PPAR-α was elevated after ICG001 treatment. Fenofibrate-induced activation of PPAR-α in vivo and in vitro reduced cardiac hypertrophy^49^. In a pressure overload model, fenofibrate lowered plasma cholesterol and glucose levels, suggesting that it could also alleviate cardiac hypertrophy by lowering myocardial lipid and glucose metabolism^49^. Moreover, by chelating the iron, RPE cells were treated with the PPAR-agonist fenofibrate, which blocked iron-induced oxidative stress and Wnt/β-catenin signaling^50^. Activating PPAR-α transcription reportedly improves the function and energetics of the heart^51^; hence, we evaluated the effect of ICG001 on metabolite concentration in the heart. One of the most noticeable metabolic changes was found in branched-chain amino acid (BCAA) metabolism. For mammals, BCAAs such as leucine, isoleucine, and valine act as important nutrient signal molecules that control cellular metabolism and growth^52^. Metabolic profiling demonstrated that amino acids including leucine, isoleucine, valine, lysine, phenylalanine, serine, tryptophan, and tyrosine were increased in TAC mice but significantly decreased in ICG001-treated TAC mice. Reducing the cardiac intratissue concentration of BCAA in TAC mice by increasing the BCAA catabolism preserves cardiac function and structure^53^; thus, stimulation of BCAA catabolism which correlated with PPAR-α upregulation may be one of the mechanisms that ICG001 protects the heart from HF. Furthermore, although ICG001 did not influence G1P and G6P, it did influence G3P, a metabolite connecting glycolysis, lipogenesis, and oxidative stress^54^, after TAC. ICG001 also reduced pyruvate, alanine, and lactate levels. A previous study showed that the alanine and lactate levels were increased in TAC mice but not in sham, suggesting reduced flux of pyruvate into the TCA cycle with diversion to lactate^55^. Metabolite analysis also revealed that TAC mice had significantly increased aspartate levels after 1, 2, or 4 weeks, which was correlated with cardiac hypertrophy^56,57^. Our results showed that alanine, lactate, and aspartate accumulated in TAC mice but this accumulation was reduced after ICG001 treatment. The attenuation of lactate, alanine and aspartate accumulation, which was correlated with ICG001 injection, may contribute to the prevention of cardiac hypertrophy and HF.

We used the GSH/GSSG ratio to monitor the redox status^58^, with higher ratios indicating reduced oxidative stress and lower ratios indicating increased oxidative stress. The GSH/GSSG ratio was significantly higher in the ICG001-treated TAC mice than in the vehicle-treated TAC mice. Hence, the oxidative stress was increased after TAC but was subsequently reduced by ICG001 injection. Thus, ICG001 may mediate the distinct alteration of oxidative stress during myocardial hypertrophy and HF. The results of metabolic flexibility in ICG001-treated TAC mice highlight the need to increase our understanding of the role and metabolism of ICG001. However, we only evaluated and reported the metabolic changes in hypertrophic and failing hearts in TAC mice in comparison with those in sham and ICG001-treated TAC mice. The metabolism of ICG001 in preventing and improving cardiac hypertrophy and HF requires further clarification in the future. Analysis of limited immune factors and only two time points, namely, before TAC and 4 weeks after TAC. Although correlations were noted, no direct links between KLF5 downregulation and proinflammatory cytokine downregulation or attenuated macrophage accumulation and between PPAR-α upregulation and metabolic alteration were observed in the present study. We also showed that ICG001 attenuated both cardiomyocyte hypertrophy and fibrosis; however, we were unable to reveal which one of two ICG001 attenuates first. Sampling at earlier time points and investigating effects of initiating ICG001 treatment after HF in future studies may reveal detailed mechanisms by which ICG001 prevents HF.

In this study, ICG001 prevented and improved the heart against hypertrophy and dysfunction by reducing the fibrosis and macrophage accumulation and induced change in substrate metabolism after pressure overload. In conclusion, ICG001 is a potential drug that may prevent cardiac hypertrophy and fibrosis through regulating KLF5, immune activation, and the Wnt/β-catenin signaling pathway and also inhibiting the inflammatory response involving the macrophages.

## Materials and methods

### Animal and pressure overload models

This study utilized 8-week-old male mice (C57BL/6 commercially purchased from Takasugi Experimental Animal Supply Co., Ltd., Japan). The Institutional Animal Care and Concern Committee at Jichi Medical University approved all selected mice, which were used according to the committee’s guidelines. The experiments also adhered to the ARRIVE guidelines^59^. The number of mice in each experiment is indicated in the figure legends. These mice were allowed to consume food and water under a 12 h light and dark cycle in a room with controlled temperature and humidity conditions. We used TAC model to induce pressure overload in mice. Furthermore, they were randomly assigned to either TAC group or sham surgery group. Briefly, they were anesthetized with medetomidine (1 mg/ml), midazolam (5 mg/ml), and butanol tartrate (5 mg/ml) and underwent a longitudinal incision along the proximal portion of the sternum. We used a 27-gage needle for ligation to yield a narrow diameter at the aortic arch when the needle was pulled out later by 6-0 silk. Afterward, we removed the needle from the ligation area. The sham group also underwent a similar procedure but without ligation.

### Echocardiography

Echocardiography was performed before and at 4 weeks after TAC or sham surgery. Briefly, all mice were placed on the heating plate in a supine position, with the extremities tied to the plate by four electrocardiography leads. The fur on the chest was removed using a hair remover cream, and the visibility of the cardiac chambers was improved by applying an ultrasound gel on the thorax area. After the mice were anesthetized with 5% of induction phase and 1.5% of maintenance phase of isoflurane, echocardiography was performed using Vevo2100 equipped with a 30 MHz linear transducer. For data collection, we measured the following factors: interventricular septum depth at end-diastole (IVSd), interventricular septum depth at end-systole (IVSs), left ventricular internal diameter at end-diastole (LVIDd), left ventricular internal dimension at end-systole (LVIDs), left ventricular posterior wall depth at end-diastole (LVPWd), left ventricular posterior wall depth at end-systole (LVPWs), left ventricular end-diastolic volume (LVEDV), left ventricular end-systolic volume (LVESV), left ventricular mass (LVM) (corrected), left ventricular ejection fraction (LVEF), left ventricular fractional shortening (LVFS), and heart rate (HR). We calculated the EF and captured its images to measure wall thickness. Measurements and analyses were conducted by two individuals who were blinded to the experimental groups of mice. Then, the mice were sacrificed to collect their blood and heart tissue samples.

### Treatments

After TAC surgery, the mice were randomly separated into two groups, namely, vehicle group (negative control) and ICG001-treated TAC group. ICG001 (50 mg/kg/day) was intraperitoneally administered for 10 days (twice per day) to evaluate its effect on cardiac function. For the vehicle group, vehicle (DMSO) was intraperitoneally administered into the following subgroups: sham and wild-type groups. EF was measured again at 4 weeks after the completion of administration. Then, we collected the heart and blood samples and measured the body weight to compare the HW/BW ratio (mg/g). Thereafter, the LV tissues were collected for further experiments.

### Histology

Heart samples were collected at indicated time points after TAC surgery. After phosphate-buffered solution (PBS) perfusion through the LV, the hearts were washed by cold PBS and fixed for 24 h in a rapid fixative solution (Sakura Finetek Japan Co., Ltd.). Tissues were embedded in paraffin and sliced into sections (5 μm). The sections were transferred onto microscope slides and stained with HE to evaluate the cardiomyocyte cross-sectional area and MT to assess the extent of fibrosis. Briefly, 5 μm-thick sections were deparaffinized and rehydrated. We used the standard method for HE staining. The sections were incubated in Carrazi’s hematoxylin solution (Muto Pure Chemicals, Ltd., Tokyo, Japan) for 5 min and washed in tap water. Then, they were differentiated in 1% acid alcohol and dehydrated in 95% ethanol, followed by incubation in eosin solution (Wako Pure Chemical Industries, Ltd) for 3 min and dehydration in a series of ethanol (80%, 95%, and 100%). Finally, they were cleared in xylene for 10 min and mounted with VectaMount (Vector). Histopathological features of each section were examined under the Keyence BZ-9000 microscope to show the pathological change of heart size and fibrosis. Cardiomyocyte cross-sectional areas (CSA) were measured according to the methods described previously^60^. Briefly, each section of hearts was photographed under a microscope (magnification 40X). Five fields were randomly selected from the left ventricle of each animal. Within each field, at least ten cells were measured in each section to calculate the cadiomyocyte CSA. The CSA and area of fibrosis were analyzed by ImageJ.

### Immunohistochemistry

We sliced 5 μm-thick sections of paraffin-embedded hearts and transferred them onto microscope slides. These sections were deparaffinized and rehydrated using xylene and graded alcohol series and then washed for 5 min in tap water. Next, they were heated in citric acid in a microwave for 8 min. The tissues were subsequently quenched with BLOXALL blocking solution for 10 min and washed in PBS buffer for 5 min. The sections were processed using the VECTASTAIN Elite ABC-HRP Kit Peroxidase (Rabbit IgG) (Vector Labs, Cat. PK-6101) and ImmPACT DAB peroxidase substrate (Vector) following the manufacturer’s protocol. Briefly, these sections were blocked with diluted normal goat serum for 20 min to block nonspecific binding. Next, some sections were incubated overnight with anti-CD68 antibody (Abcam, Cat. Ab125212) at 1:1,000 dilution to detect macrophage accumulation, while the others were incubated overnight with anti-CD3 antibody (SP7) (Abcam, Cat. Ab16669) at 1:500 dilution to detect T cell accumulation. Furthermore, the sections were washed with PBS buffer for 5 min and incubated with diluted biotinylated secondary goat anti-rabbit IgG for 30 min. Next, they were washed again with PBS buffer for 5 min and then incubated with VECTASTAIN Elite ABC reagent for 30 min, followed by another 5 min wash with PBS buffer. These sections were applied with ImmPACT DAB peroxidase substrate (Vector) for 2 min and then rinsed with distilled water. Afterward, they were counterstained with hematoxylin for 4 min, washed with distilled water, dehydrated with 70%, 80%, 90%, and 100% ethanol, cleared in xylene, and mounted in VectaMount (Vector). Images were captured using BZ-9000 (KEYENCE). The intensity of macrophage and T cell was analyzed by ImageJ.

### RNA extraction and quantitative real-time polymerase chain reaction (PCR)

After PBS perfusion through the LV, heart tissues were collected and washed in cold PBS following TAC. We extracted RNA from the LV tissues of mice by using RNeasy mini kit (QIAGEN, Cat. 74106) and removed DNA contamination by RNase-Free DNase digestion (QIAGEN, Cat. 79254) in accordance with the manufacturer’s protocol. The RNA was washed and eluted, and RNA concentration and purification were assessed with Nanodrop1000 (Thermo Fisher Scientific). According to the manufacturer’s protocol, cDNA was synthesized by ReverTra Ace reverse transcriptase (Toyobo). Real-time PCR was conducted using the SYBR Premix Ex Taq II Kit (RR820A; Takara Biotechnology) and the Stratagene Mx3005P QPCR System (Agilent Technologies, Santa Clara, California, USA). First, in the total reaction system, 25 µl was added to 2 SYBR Premix Ex Taq II (12.5 µl), cDNA (2 µl), 10 µmol/L concentration of forward primer (1 µl), 10 µmol/L concentration of reverse primer (1 µl), and RNase-free water (8.5 µl). Moreover, the mixtures were denatured at 95 °C for 30 s, processed to 40 cycles of amplification at 95 °C for 5 s, and annealed at 60 °C for 30 s. Real-time PCR was run in 96-well plates, and the relative expression levels of the target genes were evaluated after normalizing against the GAPDH gene and quantified using the comparative threshold cycle method. Table 2 lists the specific gene primer sequences.

**Table 2:** Primers for quantitative real time polymerase chain reaction.

Gene	Forward primer (5′–3′)	Reverse primer (5′–3′)
BNP	ATGGATCTCCTGAAGGTGCTG	GTGCTGCCTTGAGACCGAA
β-MHC	CCGAGTCCCAGGTCAACAA	CTTCACGGGCACCCTTGGA
CTGF	TGTGTGATGAGCCCAAGGAC	AGTTGGCTCGCATCATAGTTG
Collagen I	TGGCCTTGGAGGAAACTTTG	CTTGGAAACCTTGTGGACCAG
Fibronectin	CGAGGTGACAGAGACCACAA	CTGGAGTCAAGCCAGACACA
GAPDH	GGTGCTGAGTATGTCGTGGA	ACAGTCTTCTGGGTGGCAGT
IL-4	GGTCTCAACCCCCAGCTAGT	CCGATGATCTCTCTCAAGTGAT
IL-10	GATGCCCCAGGCAGAGAA	CACCCAGGGAATTCAAATGC
TGF-β	CTCCCGTGGCTTCTAGTGC	GCCTTAGTTTGGACAGGATCTG
TNF-α	CAGCCGATGGGTTGTACCTT	GGCAGCCTTGTCCCTTGA
Ccl2	GTCTGTGCTGACCCCAAGAAG	TGGTTCCGATCCAGGTTTTTA
Wnt1	GCCCTAGCTGCCAACAGTAGT	GAAGATGAACGCTGTTTCTCG
Wnt3a	TTCTTACTTGAGGGCGGAGA	CTGTCGGGTCAAGAGAGGAG

### Western blotting

Using T-PER tissue protein extraction reagent (Thermo Scientific, Cat. 78510), we extracted proteins from the cardiac tissues. Briefly, the protease inhibitors (Thermo Scientific Halt Protease Inhibitor Cocktail and EDTA-Free) were added to the T-PER reagent before use. The appropriate amount of T-PER reagent was added to the tissue sample and then homogenized. Thereafter, the sample was centrifuged at 10,000×*g* for 5 min to cell pellets. Next, we collected the supernatant and quantified the protein concentration by using Pierce BCA Protein Assay Kit (Thermo Scientific, Cat. 23225) according to the manufacturer’s protocol. Protein samples were mixed with 5X loading buffer and denatured in Thermo Alumi Bath (Iwaki, ABL-121) for 10 min. Then, they were cooled and stored at − 20 °C for further study. Equal amounts of protein from each sample and Precision Plus Protein Dual Color Standards marker (Bio-Rad Laboratories, Cat. 1610374) were fractionated by sodium dodecyl sulfate polyacrylamide gel electrophoresis (SDS-PAGE). The proteins were transferred from SDS-PAGE to the membrane by a gel-transfer device. Afterward, the membrane was incubated with different primary antibodies for 1 h and then with secondary antibodies for another 1 h, at room temperature. The blots were scanned with an infrared imaging system to quantify the expression of protein. The protein expression levels were normalized to the corresponding GAPDH (Thermo Scientific, Cat. AM4300) levels. The whole blot membranes corresponding to the cropped blots are shown in Supplementary Figs. 2–11).

### Flow cytometry

Heart tissues were minced and then digested with collagenase type IV (2 mg/ml, Worthington Biochemical Corporation) and Dispase II (1.2 U/ml, Sigma-Aldrich) in Dulbecco’s PBS (DPBS) supplemented with CaCl_2_ (0.9 mmol/l). Subsequently, these heart tissues were incubated at 37 °C for 15 min with gentle shaking. After incubation, these heart tissues in digestion buffer were triturated by 10 ml serological pipette for 10 times. They were again incubated at 37 °C for 15 min, triturated twice more, and then placed on ice. The cell suspensions were then filtered by 40 µm cell strainer. Filtered suspensions were added with 30 ml of DPBS in 50 ml tubes and centrifuged to collect the cells at 2,500 rpm for 20 min. Then, cell pellets were resuspended in 250 µl of 2% FCS/HBSS solution and blocked with the CD16/32 antibody (BioLegend, TrueStain FcX) at 4 °C for 1 h. Next, the cells were stained with the following primary antibodies in FACS buffer: anti-mouse CD45 antibody (BioLegend, Alexa Fluor 700), anti-mouse Ly-6C antibody (BioLegend, FITC), anti-mouse/human CD11b antibody (BioLegend, Pacific Blue), anti-mouse F4/80 antibody (BioLegend, PE/Cyanine7), and anti-mouse CCR2 APC-conjugate antibody (R&D systems). These cells were then incubated at 4 °C for 30 min in the dark. Finally, flow cytometry analysis was performed on Sony SH800 flow cytometer together with FlowJo (FlowJo, LLC). Gating strategies are detailed in Supplementary Fig. 12.

### Mass spectrometry

Heart samples from each group were randomly selected for proteomic analysis. These samples were removed from the mice and immediately frozen in liquid nitrogen at − 80 °C until use. We homogenized each heart sample (30–50 mg) in 500 μl of inner standard for liquid chromatography–mass spectrometry (LCMS) and then added 250 μl of ultrapure water. Afterward, we collected the homogenized samples, added 400 μl of chloroform, and mixed them thoroughly. The homogenization was centrifuged at 15,000 rpm for 15 min at 4 °C, and the supernatant was collected and filtered by 0.5 ml Amicon Ultra Centrifugal filters 3K. Then, the supernatant was centrifuged at 15,000 rpm for 90 min at 4 °C. Thereafter, the filtrate was lyophilized by TAITEC Ve-125 Centrifugal Concentrator overnight. Then, we added all samples with 100 μl of ultrapure water and processed through LCMS using the LCMS 8030 and 8050 (Shimadzu).

### Statistical analysis

The statistical significance of distributed data was analyzed by one-way analysis of variance (ANOVA) and two-way ANOVA followed by Tukey’s multiple comparisons test. The statistical test used in each experiment is indicated in the figure legends. All analyses were computed using the GraphPad Prism 6 software. Furthermore, *P* < 0.05 was considered significant.

## Supplementary Information

Supplementary Information.

## Data Availability

The data sets generated and/or analyzed during the current study are available from the corresponding author on reasonable request.
