# Supplementary figures and images for: Inhibition of the canonical Wnt signaling pathway by a β-catenin/CBP inhibitor prevents heart failure by ameliorating cardiac hypertrophy and fibrosis
Source: Sci Rep. 2021 Jul 21;11:14886. doi: 10.1038/s41598-021-94169-6 (PMC8295328; doi:10.1038/s41598-021-94169-6)

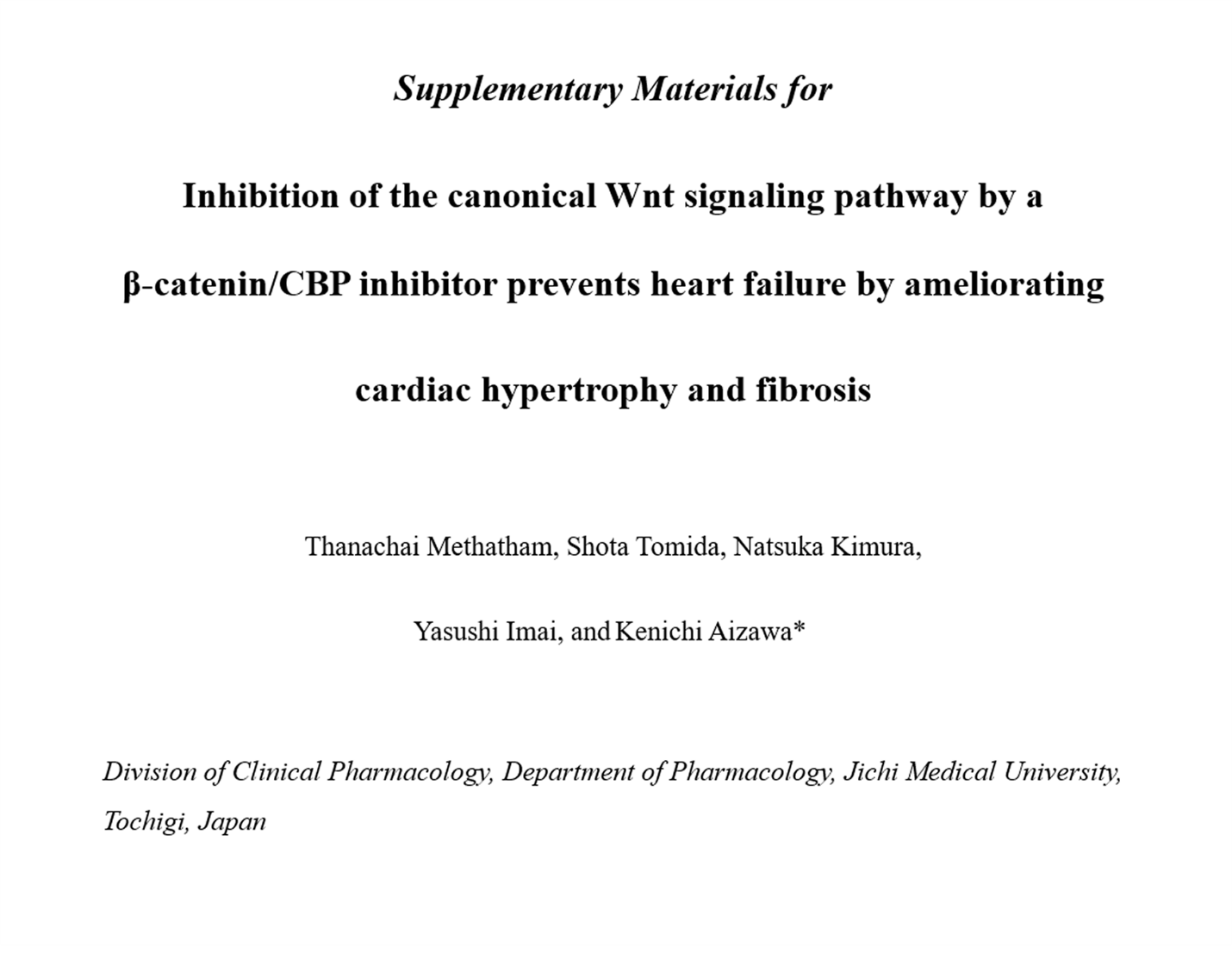


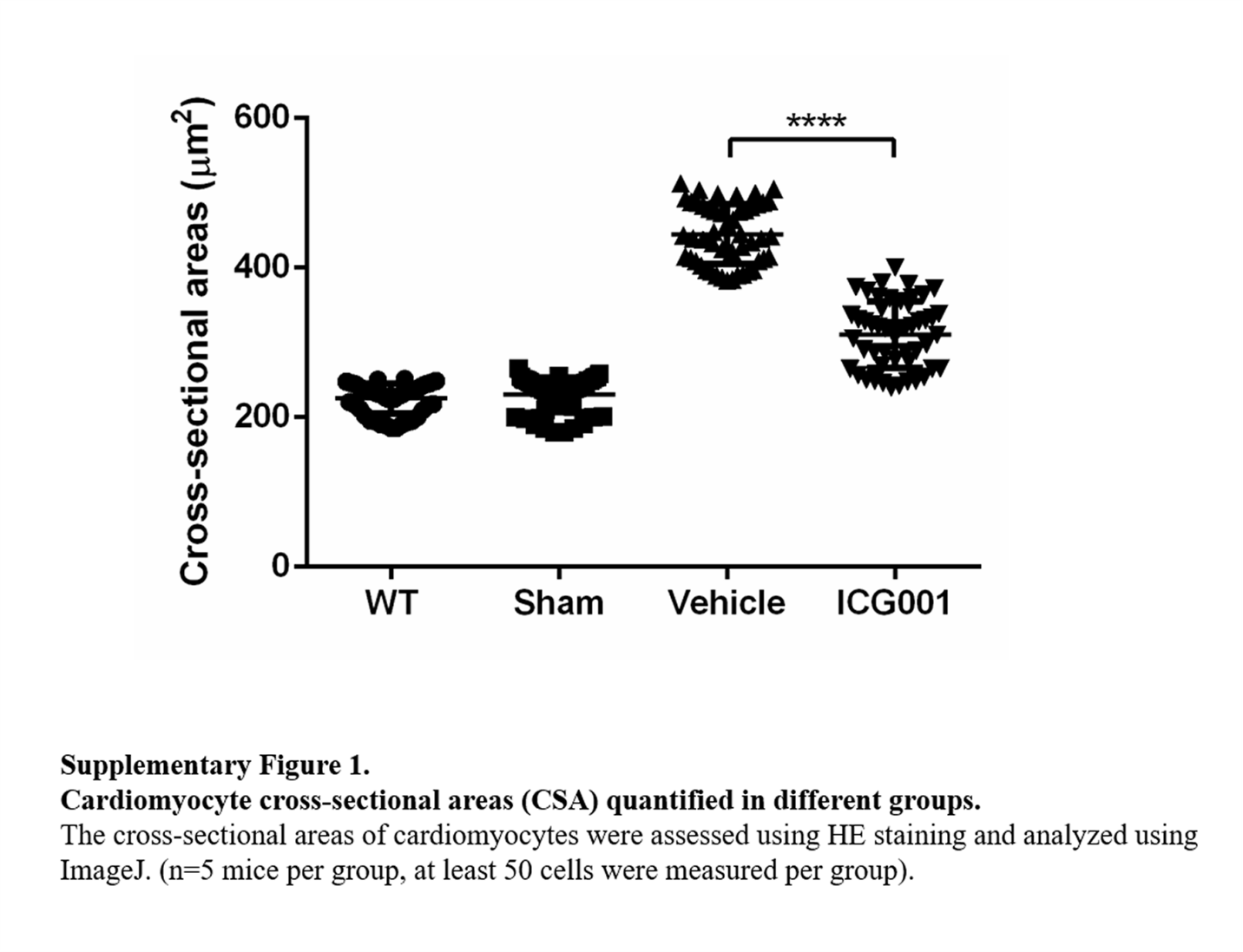


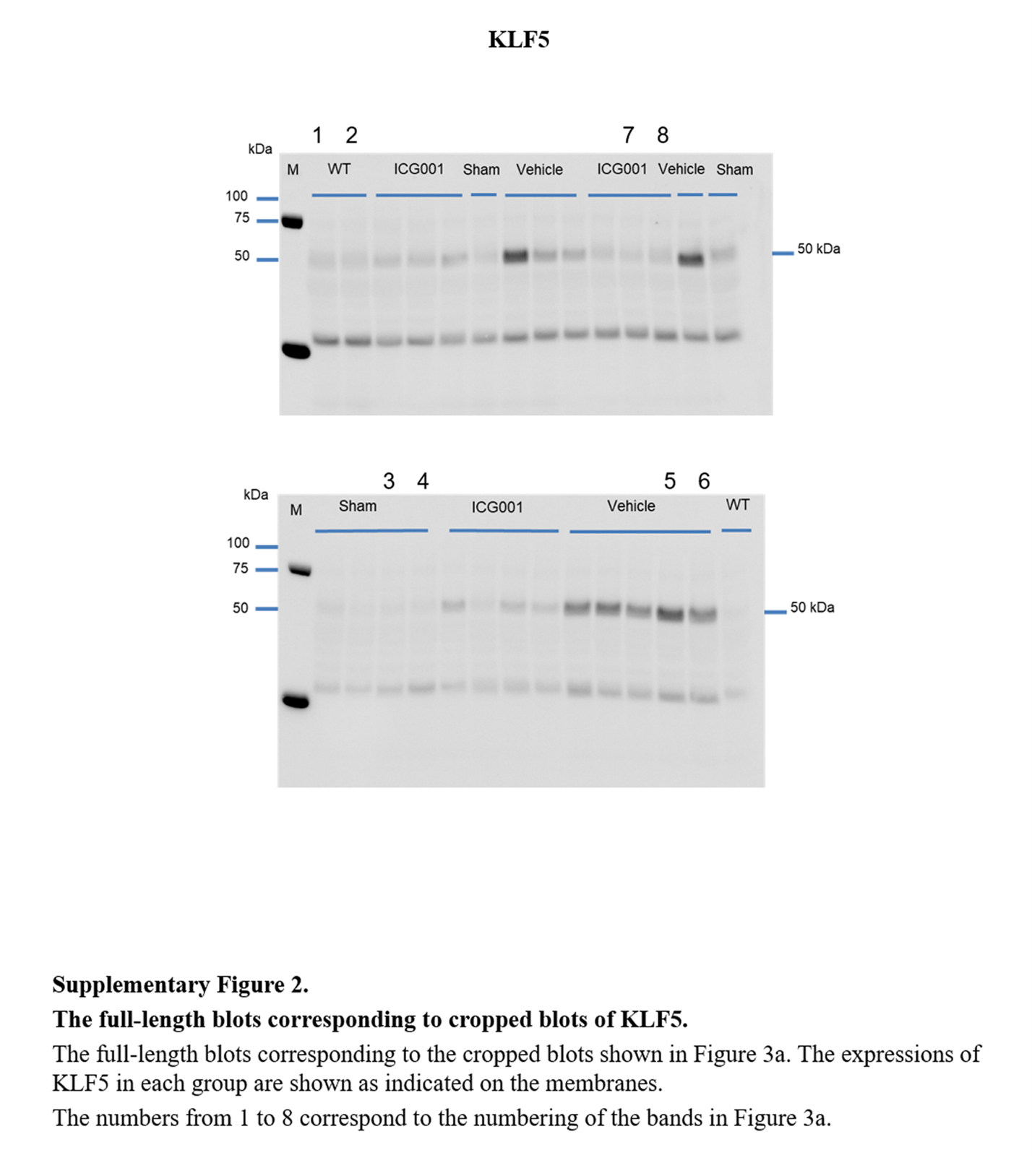


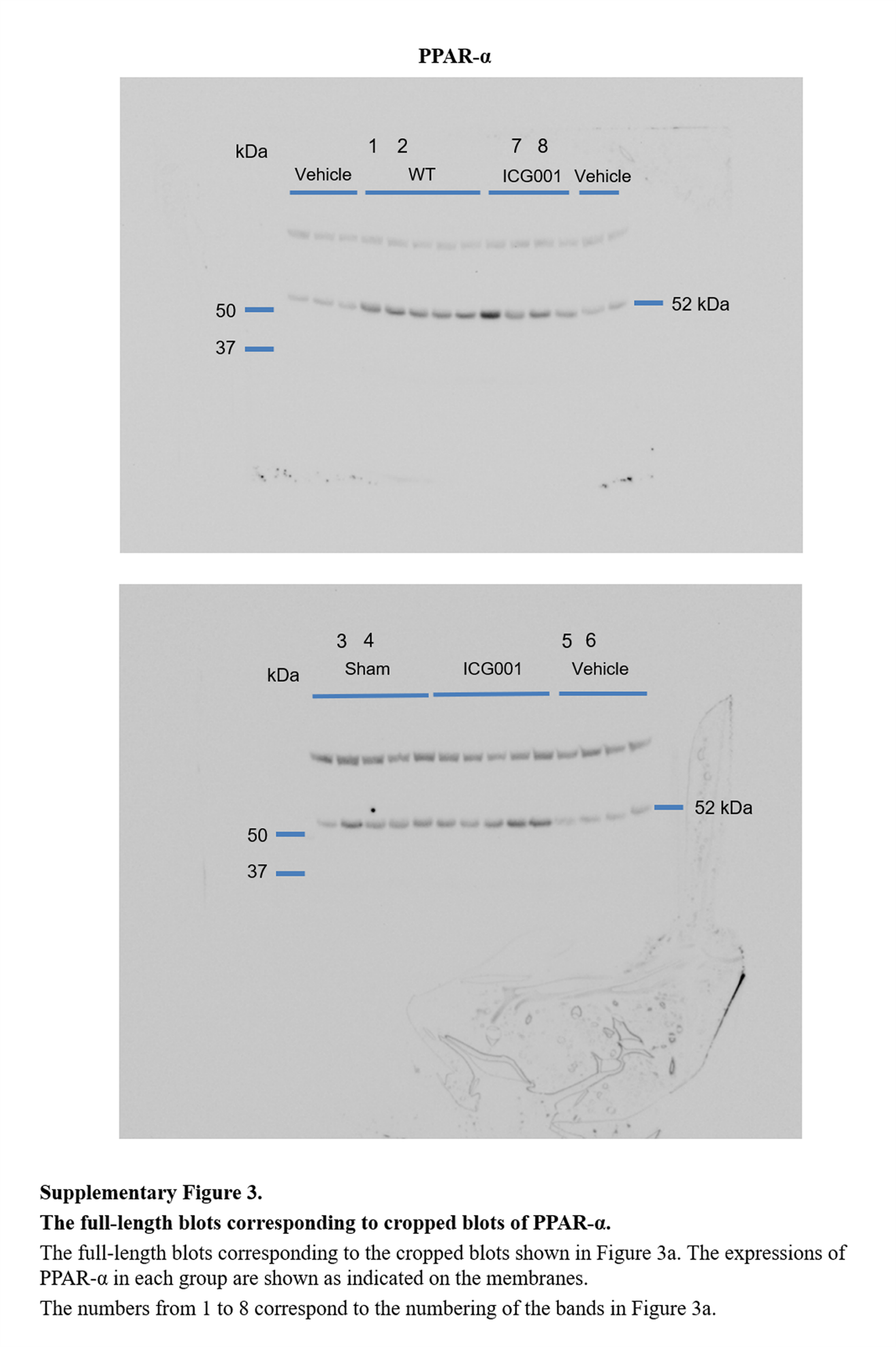


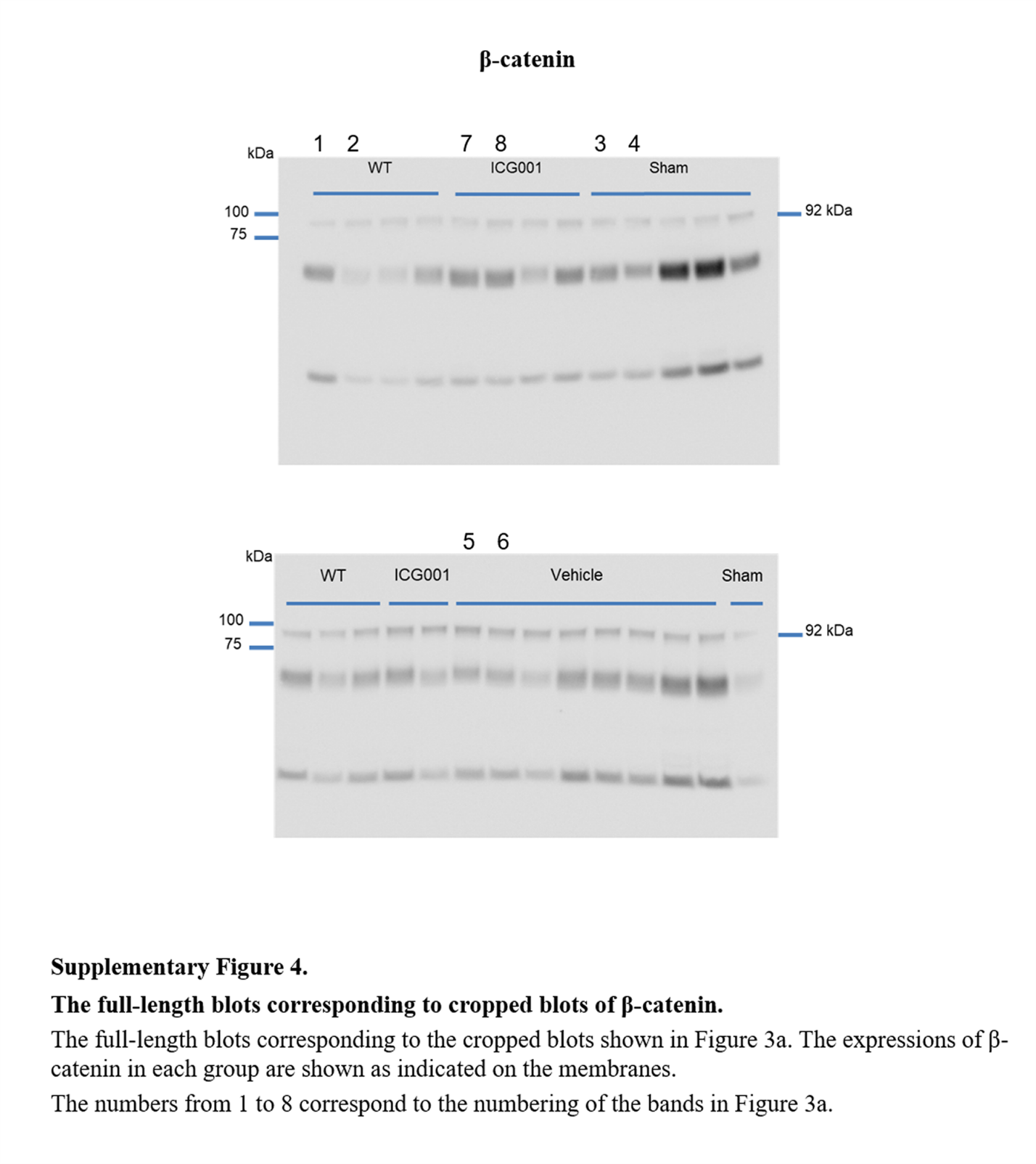


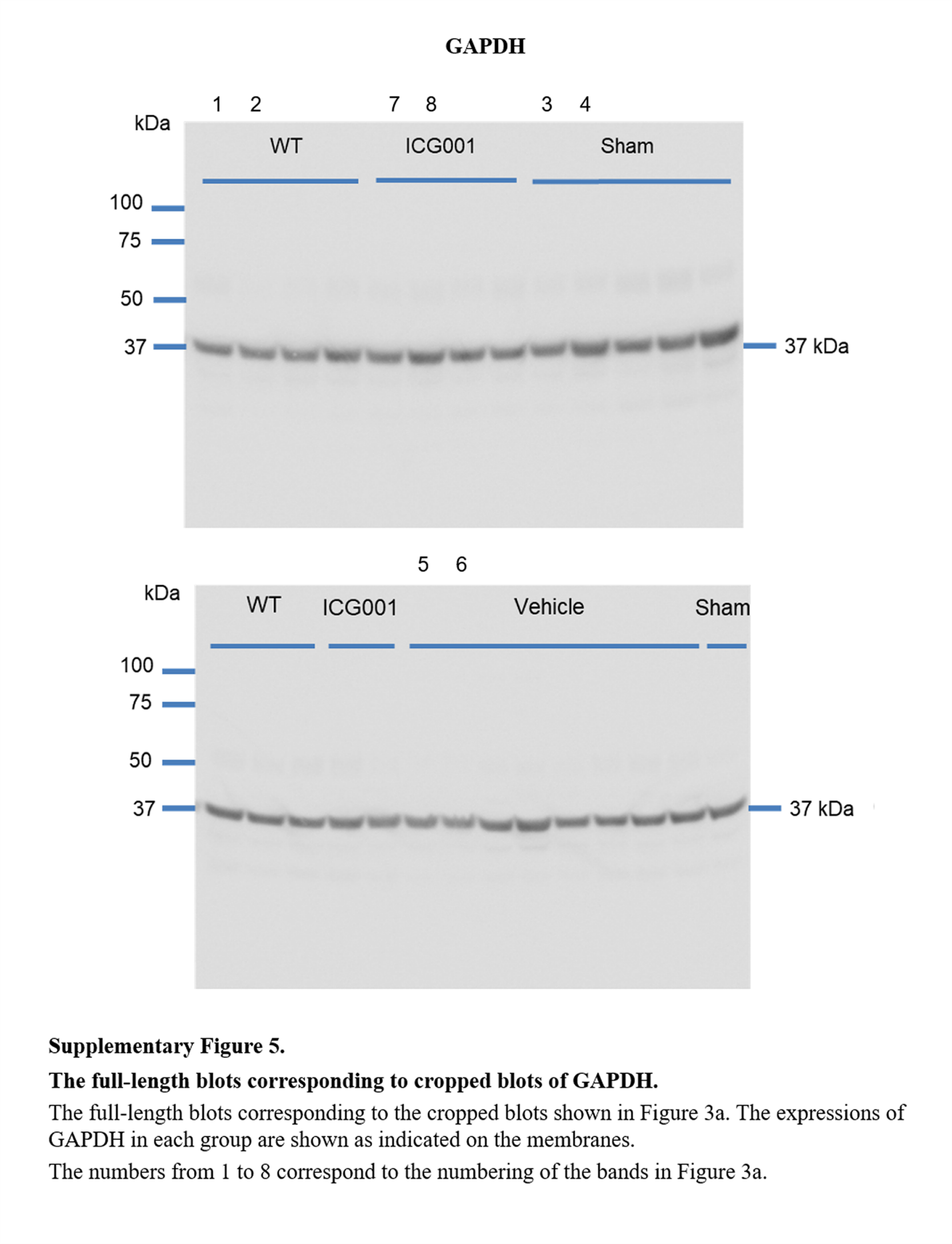


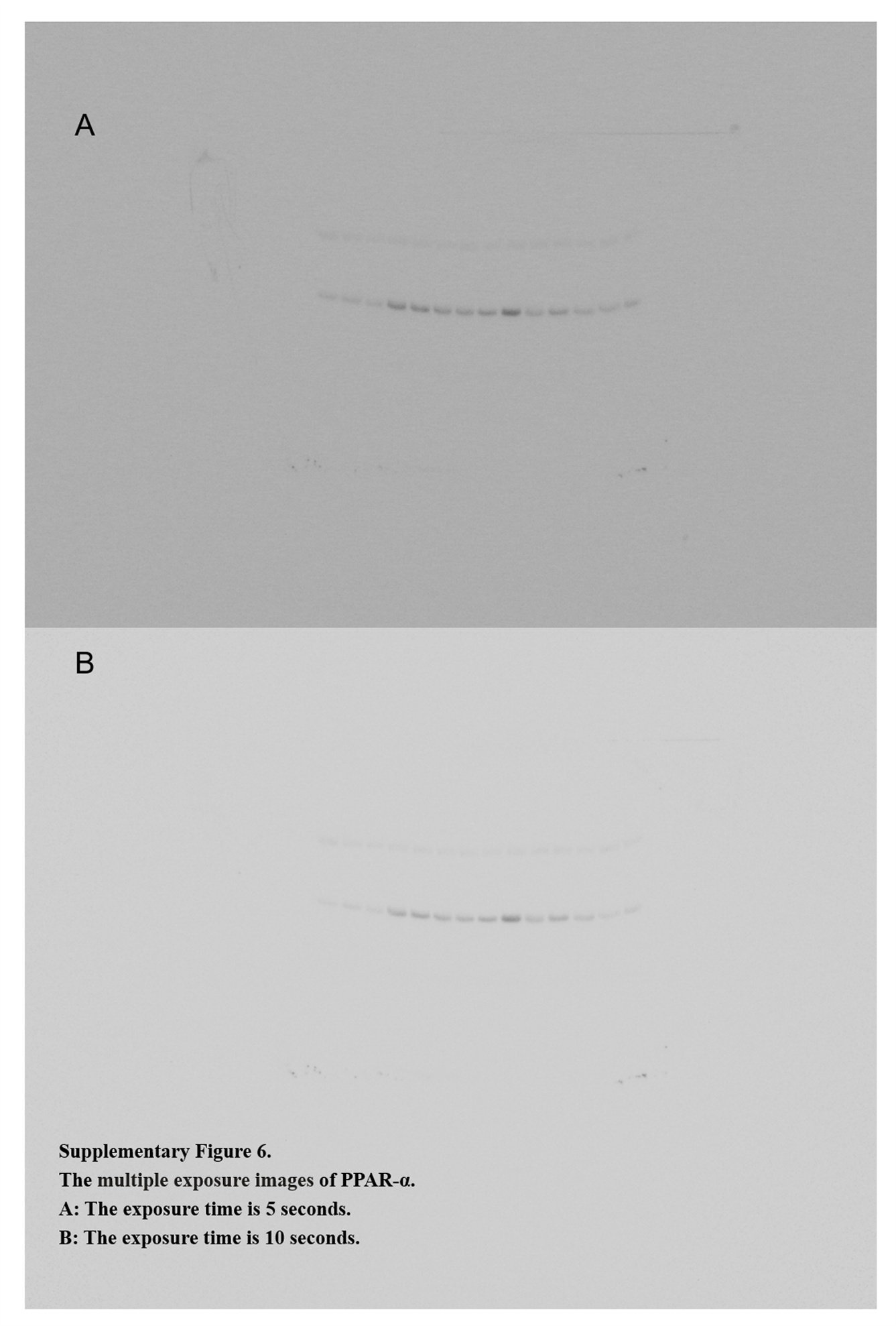


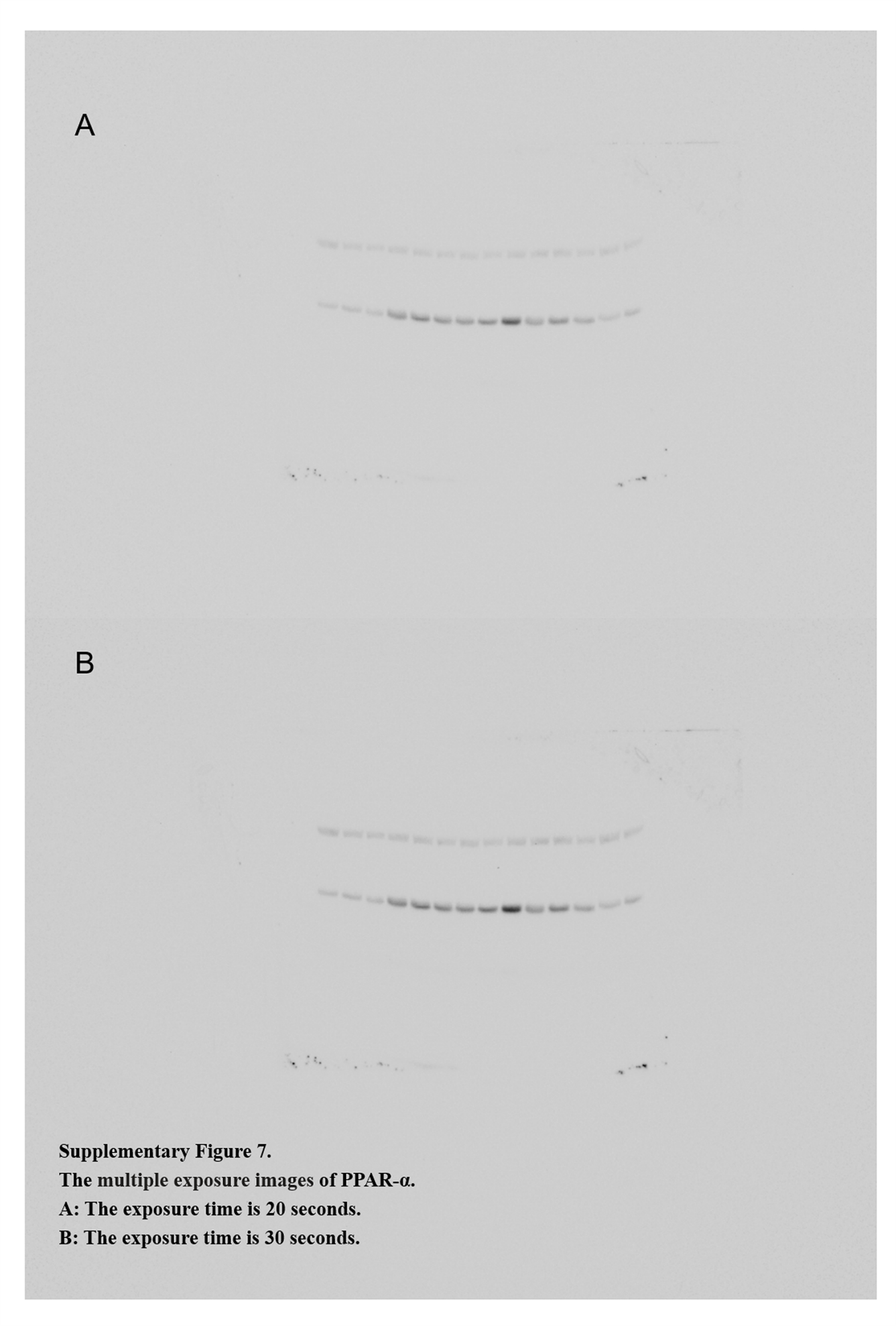


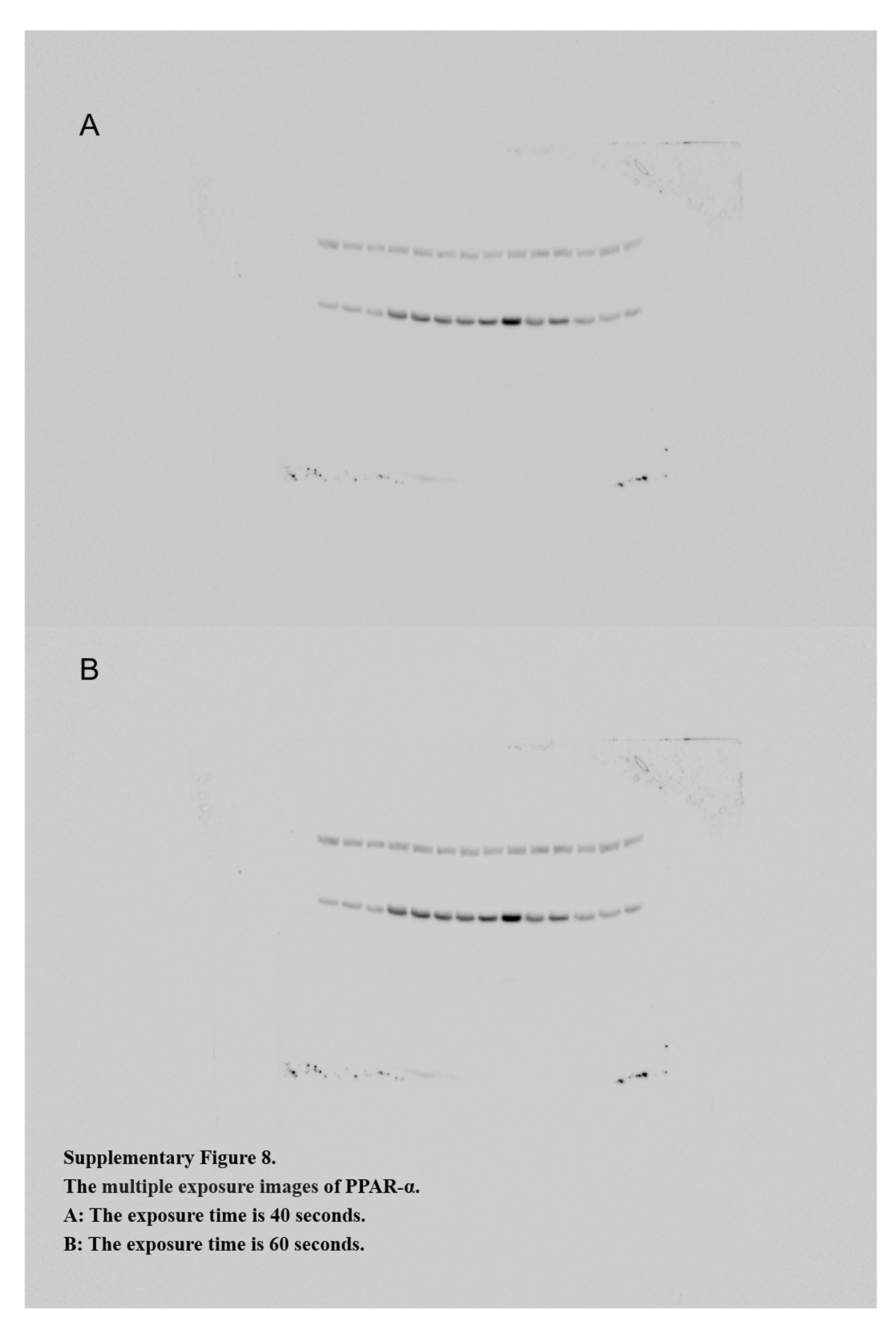


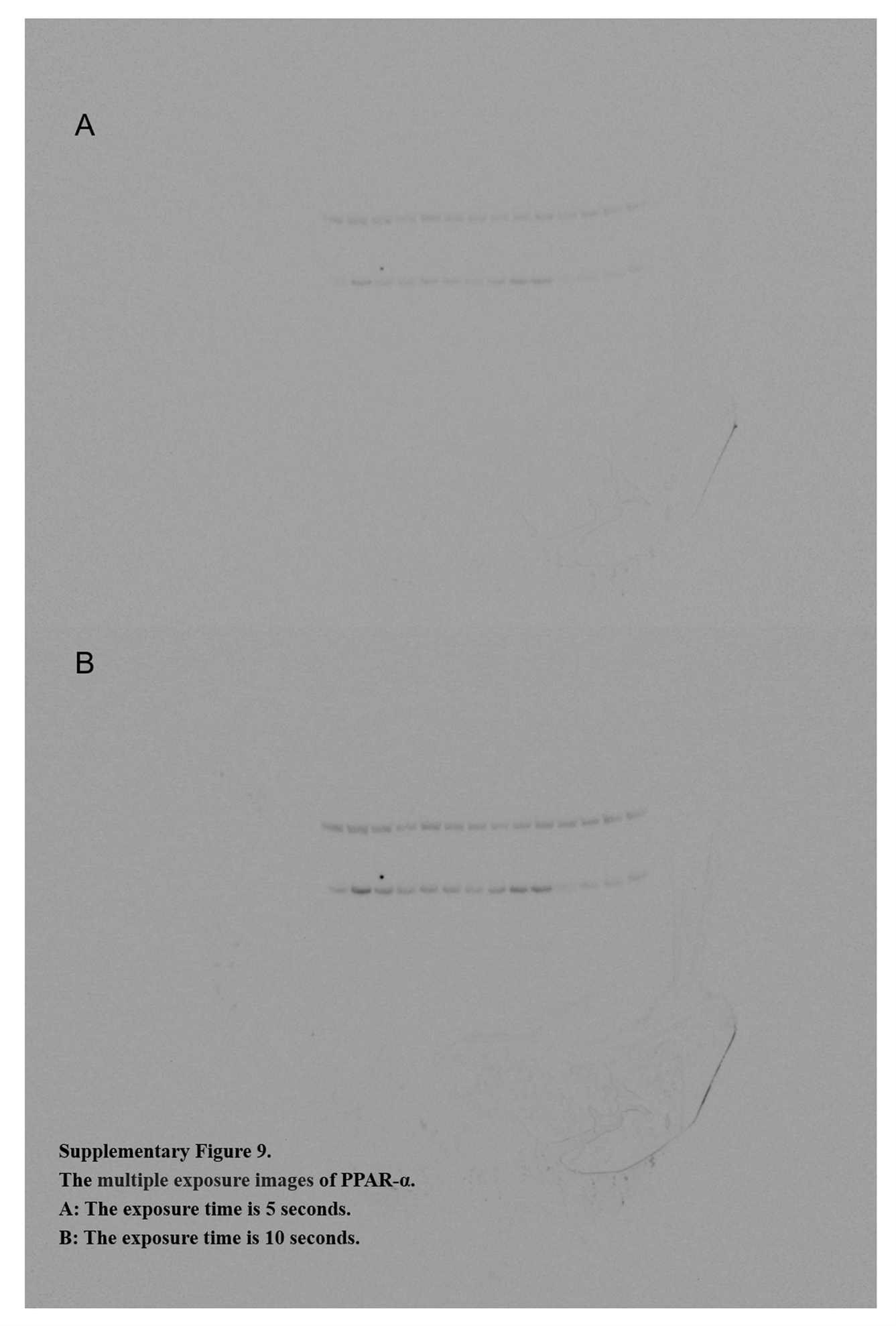


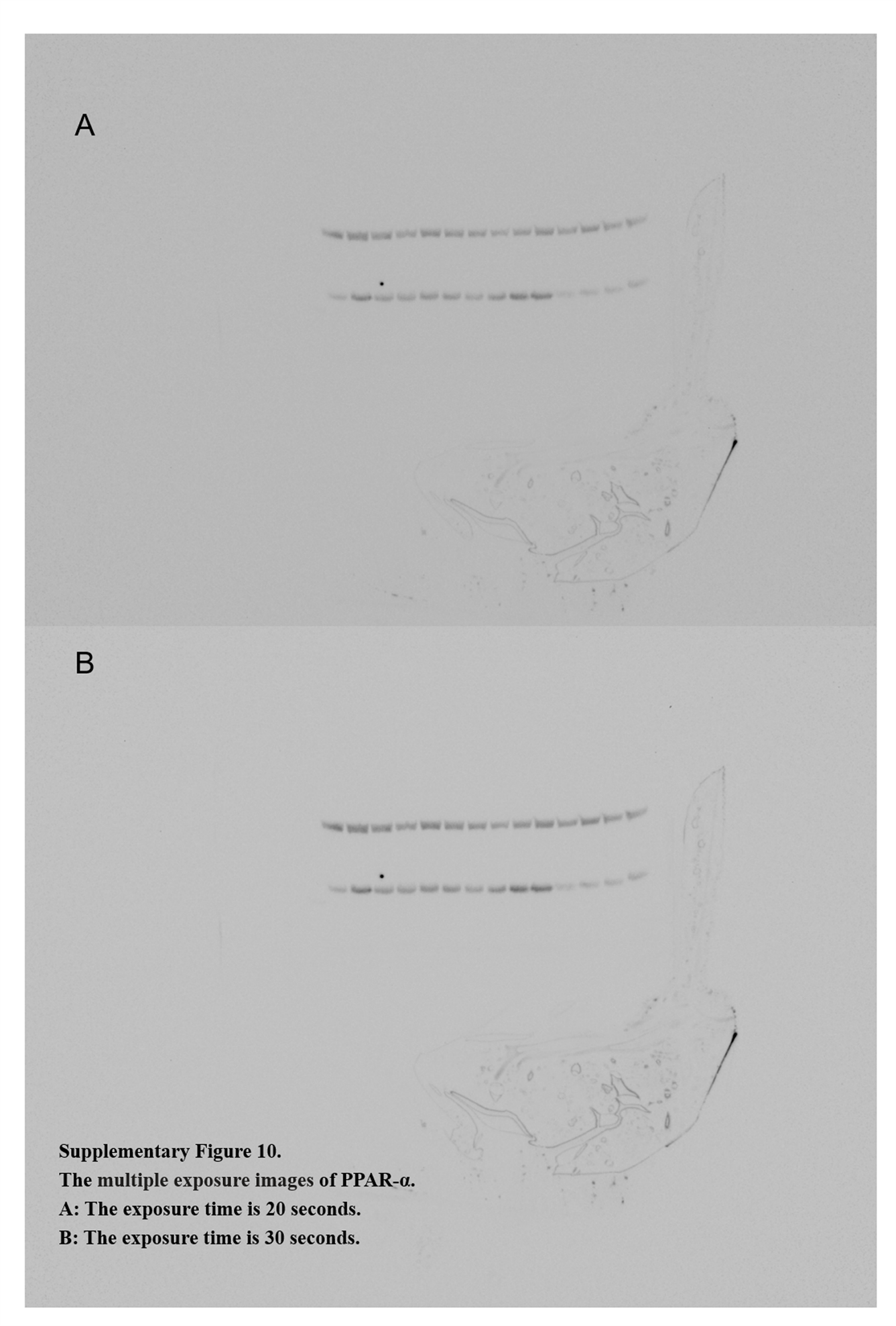


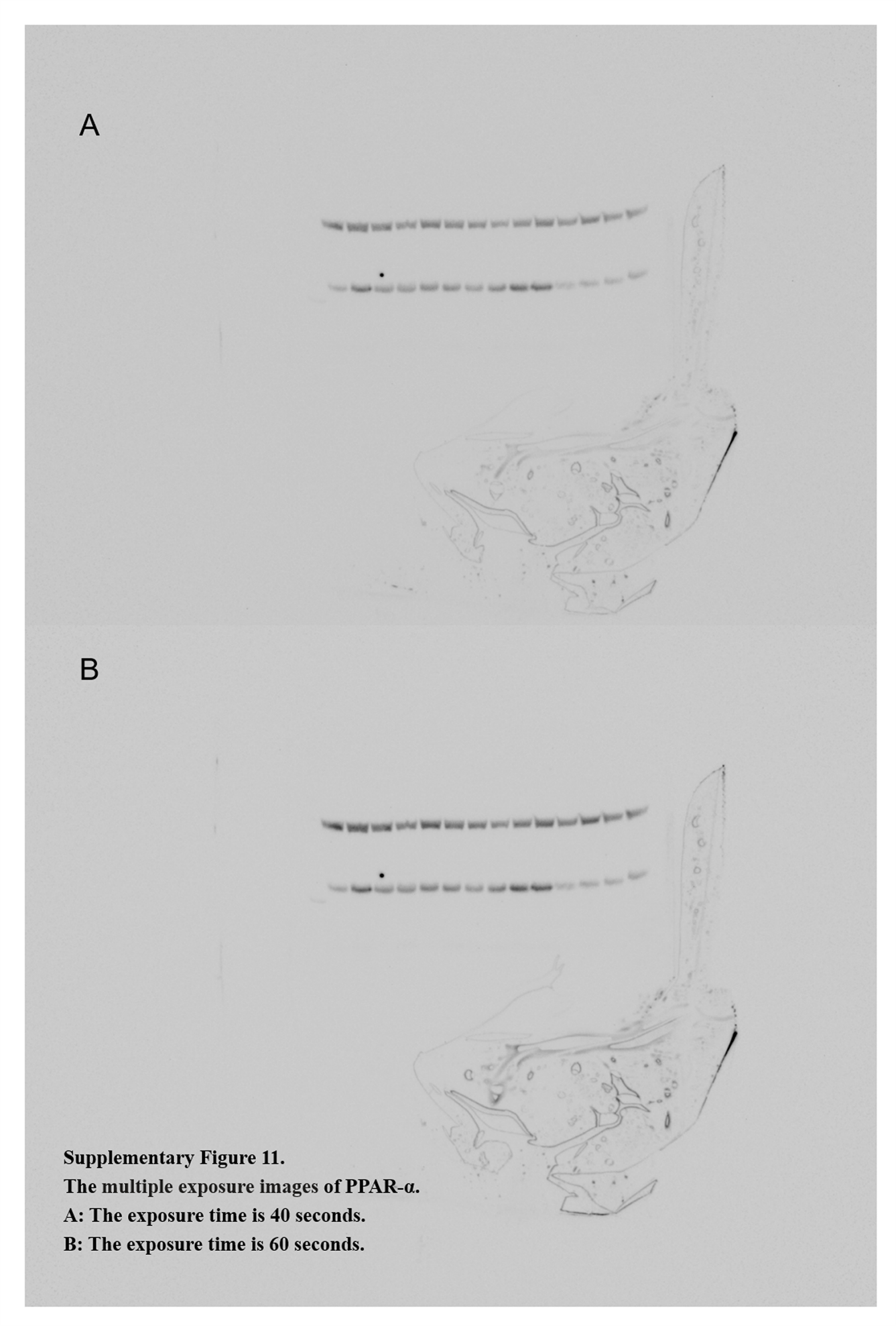


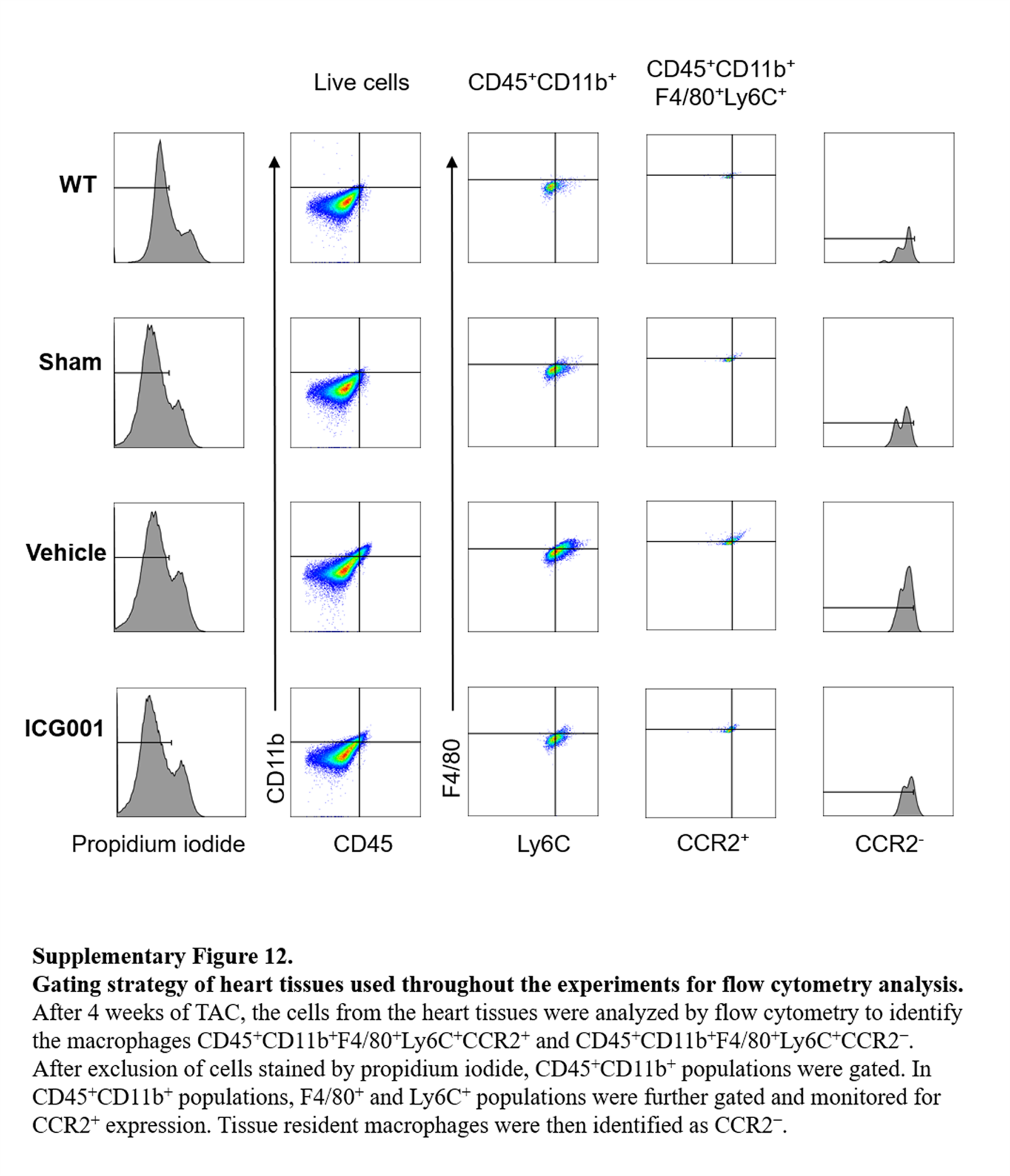

Supplement: Supplementary file 1 — Supplementary Information. [file 41598_2021_94169_MOESM1_ESM.docx]
